# A modified serine cycle in *Escherichia coli* coverts methanol and CO_2_ to two-carbon compounds

**DOI:** 10.1038/s41467-018-06496-4

**Published:** 2018-09-28

**Authors:** Hong Yu, James C. Liao

**Affiliations:** 1 Department of Chemical and Biomolecular Engineering, University of California, Los Angeles, CA 90095 USA; 2 UCLA-DOE Institute of Genomics and Proteomics, 420 Westwood Plaza, Los Angeles, CA 90095 USA; 3 Academia Sinica, 128 Academia Road, Section 2, Taipei, 115 Taiwan, ROC

## Abstract

Microbial utilization of renewable one-carbon compounds, such as methane, methanol, formic acid, and CO_2_, has emerged as a potential approach to increase the range of carbon sources for bioproduction and address climate change issues. Here, we modify the natural serine cycle present in methylotrophs and build an adapted pathway for *Escherichia coli*, which allows microorganism to condense methanol (or formate) together with bicarbonate to produce various products. We introduce the modified cycle into *E. coli* and demonstrate its capability for one-carbon assimilation through growth complementation and isotope labeling experiments. We also demonstrate conversion of methanol to ethanol by utilizing the modified serine cycle in an engineered *E. coli* strain, achieving a reaction yet to be accomplished by a one-pot chemical process. This work provides a platform to utilize various renewable one-carbon compounds as carbon sources for biosynthesis through a modified serine cycle in *E. coli*.

## Abstract (web summary)

Assimilating the abundant one-carbon compounds by an industrially-relevant microorganism can broaden the substrate range and achieve reactions that are difficult for chemical process. Here the authors show a modified serine cycle can convert methanol and CO_2_ to two-carbon compounds in an engineered *E. coli* strain.

## Introduction

One-carbon (C1) compounds can be produced from various renewable sources. For example, significant amounts of methane and CO_2_ are produced from anaerobic digestion of organic wastes^1^, resulting from agriculture, animal husbandry, and food processing. Methanol is an important intermediate in utilization of methane to synthesize other feedstock chemicals^2^. Formic acid can be produced from electrochemical reduction of CO_2_ (ref. ^3^) or as a byproduct in biomass pretreatment^4^. Chemical approaches^5^ to build C–C bonds from these C1 compounds are feasible, but require high temperature, pressure, and large capital investment. Most microbes cannot utilize these C1 compounds as carbon sources, except methylotrophs. Thus, expanding the range of microorganisms to utilize various C1 compounds for bioproduction is desirable.

**Fig. 1:**
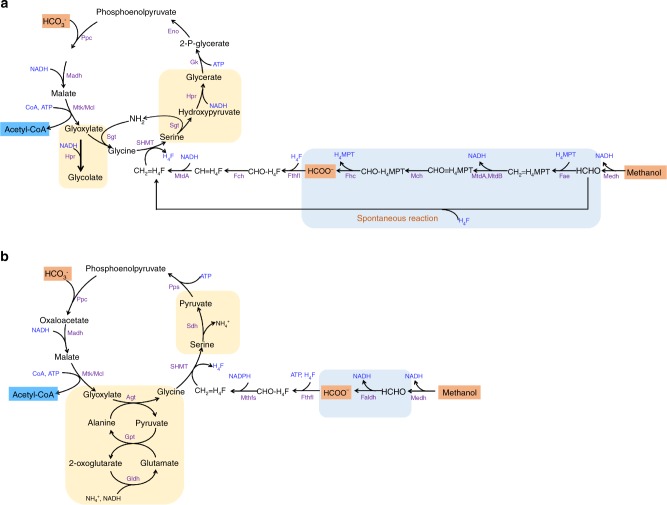
Design of the modified serine cycle for *E. coli*. **a** Illustration of the natural serine cycle in *Methylobacterium extorquens AM1*. **b** Illustration of the modified serine cycle for acetyl-CoA synthesis. Compared to the natural serine cycle (**a**), the modified serine cycle uses formaldehyde dehydrogenase (Faldh) to simplify the oxidation of formaldehyde to formate (blue box), and also utilizes the Agt Sdh combination to avoid hydroxypyruvate as an intermediate in the conversion from glyoxylate to PEP (yellow box). CH_2_=H_4_F 5,10-methylene-H_4_F, Ppc phosphoenolpyruvate carboxylase, Madh malate dehydrogenase, Mtk malate thiokinase, Mcl malyl-CoA lyase, Agt alanine-glyoxylate transaminase, SHMT serine hydroxymethyltransferase, Sdh serine dehydratase, Pps phosphoenolpyruvate synthase, Fthfl formate THF ligase, Mthfs 5,10-methylene-tetrahydrofolate synthase (C1-THF synthase), Medh methanol dehydrogenase, Faldh formaldehyde dehydrogenase, Gpt glutamate-pyruvate transaminase, Gldh glutamate dehydrogenase, Sgt serine-glyoxylate transaminase, Hpr hydroxypyruvate reductase, Gk glycerate kinase, Eno enolase, Fae formaldehyde-activating enzyme, Mtd NADP-dependent methylene-tetrahydromethanopterin/methylene-tetrahydrofolate dehydrogenase, Mch *N*(5),*N*(10)-methenyltetrahydromethanopterin cyclohydrolase, Fhc formyltransferase/hydrolase complex, Fch methenyltetrahydrofolate cyclohydrolase

The serine cycle^6^ is unique since it is the only naturally evolved oxygen-insensitive pathway that can synthesize acetyl-CoA (the C2 building block) from multiple groups of C1 compounds without carbon loss (Supplementary Table 1). Recently several papers^7–9^ have reported the construction of the ribulose monophosphate (RuMP) cycle in *E. coli* for methanol assimilation. However, the product of the RuMP cycle is dihydroxyacetone phosphate (three-carbon metabolite), which leads, when converted to acetyl-CoA, one reduced carbon is lost as CO_2_, and will cause one-third decrease in carbon yield during producing acetyl-CoA derived compounds. The synthetic methanol condensation cycle (MCC)^10^, although efficient in acetyl-CoA synthesis, has not been implemented in vivo yet. The serine cycle (Fig. 1a) uses phosphoenolpyruvate (PEP) carboxylase (Ppc) and serine hydroxymethyltransferase (SHMT) to fix bicarbonate and C1-carbon unit carried as 5,10-methylene-tetrahydrofolate (H_4_F), respectively. The carbon assimilation part of the cycle contains three segments that involve two-carbon (C2), three-carbon (C3), and four-carbon (C4) metabolites. The C2 segment includes glyoxylate, glycine, and the output acetyl-CoA. SHMT catalyzes the reaction of glycine and 5,10-methylene-tetrahydrofolate to form serine (C3), which is further converted to PEP (C3) through several intermediates including hydroxypyruvate and glycerate. Ppc performs the carboxylation of PEP to generate oxaloacetate (OAA) (C4), which is split into two C2 molecules to complete the cycle and output the product acetyl-CoA.

Here we adapt the natural serine cycle^6,11,12^ present in *Methylobacterium extorquens AM1*. We express the modified cycle in *E. coli* and demonstrate its capability for C1 compound assimilation by experiments of growth complementation and isotope labeling. The engineered strains are able to co-assimilate formate (or methanol) together with a pyruvate source, such as xylose, to improve the production of acetyl-CoA derived C2 compounds. The modified serine cycle, in principle, can support *E. coli* to grow on formate (or methanol) alone, but requires further adaptation. This platform allows *E. coli* to co-utilize multiple C1 compounds with xylose (or glucose) for bioproduction, and eventually using reduced C1 compounds only.

## Results

### Characterization of serine metabolism in *E. coli*

*E. coli* synthesizes serine through either two glycine or one 3-phosphoglycerate (Supplementary Fig. 1A). In the glycine route, one glycine is first cleaved by the glycine-cleavage (Gcv) complex to generate a C1-carbon unit carried as 5,10-methylene-H_4_F, which is then added to another glycine to produce one serine by GlyA(*E.c*) (acting as SHMT). The 3-phosphoglycerate route requires a key enzyme SerA(*E.c*), functioning as d-3-phosphoglycerate dehydrogenase^13^. Deletion of the *serA* gene makes the strain a serine auxotroph, which cannot grow in glucose minimal medium unless supplemented with serine or glycine, but not glyoxylate (Supplementary Fig. 1C), suggesting that *E. coli* cannot metabolize glyoxylate to produce glycine.

*E. coli* wild type (wt) cannot utilize serine as a sole carbon source for growth (Supplementary Fig. 1B), presumably because it is unable to convert serine into the C3 metabolite pool^14^. However, *E. coli* wt can grow in minimal medium with serine plus glyoxylate addition when heterologously expressing *sgaA* (coding for serine-glyoxylate transaminase, the key enzyme of the natural serine cycle) from *Rhodobacter sphaeroides* (Supplementary Fig. 2B). This result indicated that *E. coli* utilized hydroxypyruvate, the product of the serine transamination with glyoxylate, to generate glycerate for growth (Supplementary Fig. 2A). Two *E. coli* proteins, GhrA or GhrB, might perform the reduction of hydroxypyruvate to glycerate. However, deletion of *ghrA*, but not *ghrB*, abolished the phenotype in the same medium (Supplementary Fig. 2C), suggesting that GhrA(*E.c*) functioned as the major hydroxypyruvate reductase in vivo. By using this platform, we evaluated the efficiency of the transamination part of the natural serine cycle in *E. coli*. A previous report^15^ demonstrated that GhrA(*E.c*) also had a glyoxylate reductase activity, which exhibited 10-fold higher catalytic efficiency on glyoxylate than hydroxypyruvate. Indeed, increasing expression levels of *ghrA*(*E.c*) gradually decreased the growth rate of *E. coli* wt in minimal medium with serine and glyoxylate as carbon sources (Supplementary Fig. 2D), possibly because of its draining of glyoxylate to form glycolate irreversibly (Supplementary Fig. 2A). Therefore, we concluded that using hydroxypyruvate reductase might not be a suitable strategy to realize the natural serine cycle in *E. coli*, since its side reactivity would drain the intermediate glyoxylate, and eventually slow down the assimilation of formate (or methanol) (yellow part of Fig. 1a).

### Adapting the serine cycle in *E. coli*

As such, we chose to modify the natural serine cycle to avoid hydroxypyruvate as an intermediate, since completely removing the glyoxylate-reducing activity from hydroxypyruvate reductase was challenging in *E. coli*. In the modified serine cycle (Fig. 1b and Supplementary Table 2), instead of using serine as an amino group donor, we planned to have glyoxylate transaminated with alanine to form glycine, catalyzed by alanine-glyoxylate transaminase (Agt), followed by endogenous glutamate-pyruvate transaminase (Gpt) and glutamate dehydrogenase (Gldh) for ammonia assimilation (yellow box in Fig. 1b). Glycine is then converted to serine by addition of a C1-carbon carrier 5,10-methylene-H_4_F, derived from formate or methanol, as in the natural serine cycle. Finally, serine is deaminated to pyruvate through serine dehydratase (Sdh), and further regenerate PEP to close the cycle. Since *E. coli* does not synthesize tetrahydromethanopterin (H_4_MPT), we utilize an NAD-linked formaldehyde dehydrogenase (Faldh) to simplify the oxidation of formaldehyde to formate (blue box in Fig. 1b). Then, an H_4_F-linked pathway is used to convert formate to 5,10-methylene-H_4_F for serine synthesis.

The overall reactions of the modified serine cycle are shown in Supplementary Table 3. If acetyl-CoA is the product of the cycle, the net reaction is to convert one formate (or methanol) and one bicarbonate to produce an acetyl-CoA with the expense of 3 ATP and 3 NADH (or one NADH if methanol is used) (#1 and #2 of Supplementary Table 3). If bicarbonate is provided by formate (or methanol) oxidation, the overall reaction is to condense two formate (or methanol) to one acetyl-CoA with three ATP and two NADH consumed (or two NADH produced by using methanol) (#3 to #4 of Supplementary Table 3).

In order to produce a C3 metabolite, the pathway can be further modified to include the partial glyoxylate cycle mediated by isocitrate lyase (Icl) (Supplementary Fig. 3). In this scenario, Ppc and SHMT can fix one bicarbonate and two C1-carbon units carried as 5,10-methylene-H_4_F, respectively, to produce one pyruvate. The net reactions for the output pyruvate are shown in Supplementary Table 3 (#6 to #9). Since both the C2 and C3 building blocks, acetyl-CoA and pyruvate, can be generated, the modified serine cycle, in principle, can support *E. coli* growth on reduced C1 compounds alone, such as formate or methanol. However, growth on a reduced C1 requires a significant adaptation of cell physiology, we sought to first explore the acetyl-CoA producing mode of the modified serine cycle by allowing *E. coli* to co-utilize C1 compounds with a pyruvate source such as glucose or xylose.

### Determination of enzyme for converting glyoxylate to glycine

To establish the modified serine cycle in *E. coli*, we began by searching for suitable enzymes. Previously we demonstrated the feasibility of reversing the glyoxylate shunt in an OAA auxotrophic *E. coli* strain^16^. Introduction of malate thiokinase (Mtk) (originally annotated as SucCD-2) from *Methylococcus capsulatus* and malyl-CoA lyase (Mcl) from *Methylobacterium extorquens* in *E. coli* could achieve strong activities in splitting malate to produce acetyl-CoA and glyoxylate. Here we would focus on enzymes that could convert glyoxylate to pyruvate by condensation with one formate (or methanol).

**Fig. 2:**
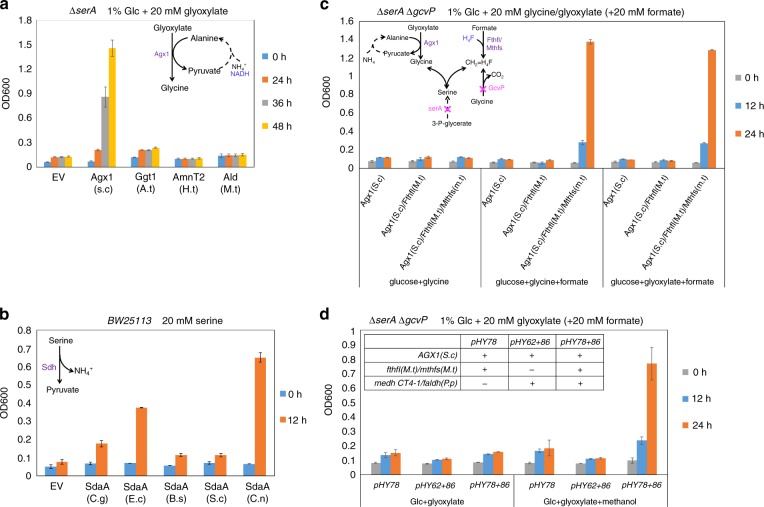
Determination of the suitable enzymes to establish the modified serine cycle in *E. coli*. **a** Expression of *AGX1*(*S.c*) allowed the Δ*serA* strain to grow in minimal medium with glucose and glyoxylate as carbon sources, suggesting Agx1(*S.c*) utilized alanine as an amino donor to convert glyoxylate to glycine and thus rescued the Δ*serA* strain. Glc glucose. **b** Expression of *sdaA*(*C.n*) enabled *E. coli* wt strain to rapidly grow in minimal medium with serine as the sole carbon source. **c** Introduction of Agx1(*S.c*), Fthfl(*M.t*), and Mthfs(*M.t*) allowed the Δ*serA* Δ*gcvP* strain to grow in glucose minimal medium with supplements of glyoxylate (or glycine) and formate. **d** Expression of *medh*(*CT4–1*) and *faldh*(*P.p*) could oxidize methanol to formate in vivo. Introduction of a set of enzymes, including Agx1(*S.c*), Fthfl(*M.t*), Mthfs(*M.t*), Medh(CT4–1), and Faldh(*P.p*), enabled the Δ*serA* Δ*gcvP* strain to grow in glucose minimal medium with supplements of glyoxylate and methanol. Error bars are s.d. (standard deviation), *n* = 3. *S.c* *Saccharomyces cerevisiae*, *A.t* *Arabidopsis thaliana*, *H.t* *Hydrogenobacter thermophiles,* *M.t* *Mycobacterium tuberculosis,* *C.g* *Corynebacterium glutamicum*, *E.c* *Escherichia coli*, *B.s* *Bacillus subtilis 168*, *C.n* *Cupriavidus necator,* *M.t* *Moorella thermoacetica, P.p Pseudomonas putida kt2440*

To select enzymes that can convert glyoxylate to glycine, a Δ*serA* strain was used (Supplementary Fig. 1C). We found that the introduction of Agx1 (alanine-glyoxylate aminotransferase 1) from *Saccharomyces cerevisiae* allowed growth of the Δ*serA* strain in minimal medium with glucose and glyoxylate supplements over 36 h (Fig. 2a). In contrast, expression of *ggt1* (from *Arabidopsis thaliana*) or *amnT2* (from *Hydrogenobacter thermophiles*), both coding for glutamate-glyoxylate transaminases, displayed much lower or even no rescuing-effect under the same conditions (Fig. 2a). These results indicated that Agx1(*S.c*) utilized alanine as an amino donor to convert glyoxylate to glycine, and thus complemented the growth defect of the Δ*serA* strain. We also tested the possibility of using glycine dehydrogenase, Ald (P9WQB0)^17^ from *Mycobacterium tuberculosis*, to directly catalyze the amination of glyoxylate to form glycine (Fig. 2a). However, the results showed that Ald(*M.t*) preferred to perform the oxidation of glycine to glyoxylate in vivo (Supplementary Fig. 4A, B).

### Utilization of serine dehydratase to produce pyruvate

For the pyruvate regeneration segment, we compared the activity of serine dehydratase^18^ from various organisms. As demonstrated above, *E. coli* wt could not utilize serine as the sole carbon source (Supplementary Fig. 1B). However, expression of *sdaA* (coding for serine dehydratase) from *Cupriavidus necator* enabled the wild-type strain to rapidly grow in minimal medium with serine addition only (Fig. 2b), suggesting that this enzyme effectively converted serine to pyruvate. More importantly, we found that its function was to catalyze the serine deamination irreversibly, since expressing *sdaA*(*C.n*) could not support the Δ*serA* strain to grow in minimal medium with pyruvate supplement (Supplementary Fig. 4C). These results indicated that the serine deamination could function as a driving force for the modified serine cycle.

### Determination of enzymes for 5,10-methylene-H_4_F synthesis

To identify enzymes catalyzing the assimilation of formic acid, we constructed a strain *ΔserA* Δ*gcvP* by deleting all the endogenous pathways^13,19^ that could produce 5,10-methylene-H_4_F in *E. coli* (Supplementary Fig. 1A, D). Then a heterologous 5,10-methylene-H_4_F synthesis pathway, catalyzed by formate-H_4_F ligase (Fthfl) and 5,10-methylene-H_4_F synthase (Mthfs), was introduced into the strain Δ*serA* Δ*gcvP*, which would allow the strain to utilize formate as a source to synthesize 5,10-methylene-H_4_F for growth. The results showed that expression of *AGX1*(*S.c*), *fthfl* and *mthfs*, both from *Moorella thermoacetica*, led the Δ*serA* Δ*gcvP* strain to grow in glucose minimal medium with supplements of glyoxylate (or glycine) and formate (Fig. 2c). The controls, with no formate addition or omitting *fthfl*(*M.t*) *mthfs*(*M.t*) expression, did not support cell growth, which demonstrated that Fthfl(*M.t*) together with Mthfs(*M.t*) were indeed able to convert formate to 5,10-methylene-H_4_F. A recent paper^20^ reported that expression of *fthfl* from *Methylobacterium extorquens* and *folD* from *E. coli* could convert formic acid to 5,10-methylene-H_4_F as well. We compared these two combinations, and showed that in our hands Fthfl(*M.t*)/Mthfs(*M.t*) displayed much higher activities in rescuing the Δ*serA* Δ*gcvP* strain in glucose minimal medium with glyoxylate/formate supplements (Supplementary Fig. 4D).

### Determination of enzymes for methanol oxidation to formate

For methanol oxidation, a variant (CT4–1)^21^ of Mdh2 (*Cupriavidus necator*) was used, which displayed the highest activity (about 0.29 μmol/min/mg of specific activity and 9.3 s^−1^ M^−1^ of *K*_cat_/*K*_m_) among the enzymes tested at 37 °C through in vitro assay (Supplementary Fig. 4E). Expressing a set of genes, including *AGX1*(*S.c*), *fthfl*(*M.t*), *mthfs*(*M.t*), *medh*(*CT4–1*), and *faldh* (coding for formaldehyde dehydrogenase) from *Pseudomonas putida*, allowed the Δ*serA* Δ*gcvP* strain to grow in glucose minimal medium with glyoxylate and methanol addition (Fig. 2d), suggesting that the Medh(CT4–1) Faldh(*P.p*) combination oxidized methanol to formate as the source for 5,10-methylene-H_4_F synthesis. Thus, we determined all the suitable enzymes needed for converting glyoxylate to pyruvate by using formate (or methanol) as a C1 donor in *E. coli*.

### Construction of the modified serine cycle in *E. coli*

To construct the modified serine cycle in *E. coli*, we first characterized the function of the cycle in overlapping segments. The protein AceA(*E.c*) acts as Icl, catalyzing the isocitrate splitting to produce glyoxylate, and its deletion abolishes the ability of *E. coli* strain to condense acetyl-CoA to form C4 metabolites through the glyoxylate shunt (Supplementary Fig. 1E). In a Δ*serA* Δ*aceA* strain, *E. coli* cannot synthesize serine from 3-phosphoglycerate or glyoxylate (Supplementary Fig. 5A). We showed that the introduction of Mtk(*M.c*), Mcl(*M.e*), and Agx1(*S.c*) rescued the growth defect of the Δ*serA* Δ*aceA* strain in minimal medium with pyruvate as the sole carbon source (doubling time of 1.7 h) (Supplementary Fig. 5B). However, as a control, expression of *AGX1*(*S.c*) alone did not display any effect, which demonstrated the critical role of Mtk(*M.c*) Mcl(*M.e*) in glyoxylate-producing in the serine cycle (marked as blue arrow in Supplementary Fig. 5A).

**Fig. 3:**
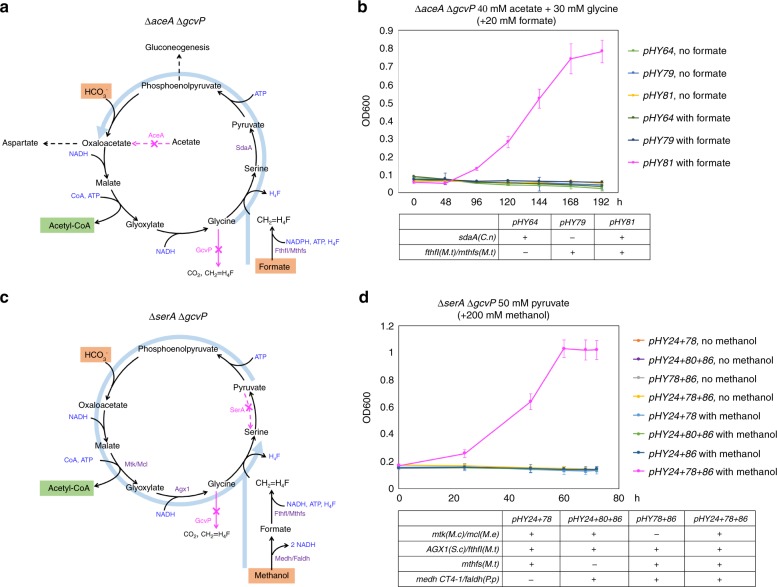
Construction of the modified serine subpathway in *E. coli*. **a**, **b** Expression of *sdaA*(*C.n*), *fthfl*(*M.t*), and *mthfs*(*M.t*) could support the Δ*aceA* Δ*gcvp* strain to grow in minimal medium by using glycine/formate/acetate as carbon sources (**b**), which indicated the function of the modified serine subpathway marked as blue arrow in **a**. **c**, **d** Expression of a set of heterologous genes, including *mtk*(*M.c*), *mcl*(*M.e*), *AGX1*(*S.c*), *fthfl*(*M.t*), *mthfs*(*M.t*), *medh*(*CT4–1*), and *faldh*(*P.p*), allowed the Δ*serA* Δ*gcvp* strain to grow in minimal medium with pyruvate and methanol as carbon sources (**d**), which demonstrated the function of the modified serine subpathway marked as blue arrow in **c**. Error bars are s.d., *n* = 3

To investigate the capability of the cycle for formate (or methanol) assimilation, an *E. coli* strain, Δ*aceA* Δ*gcvP*, was constructed. As previously described (Supplementary Fig. 1E), the *aceA*(*E.c*) deletion abolishes *E. coli* strain to condense acetate to form C4 and C3 metabolites. To rescue its growth phenotype (Supplementary Fig. 1F), part of the modified serine cycle would be applied in order to support the Δ*aceA* Δ*gcvP* strain to synthesize C3 or C4 metabolites from glycine and formate. The Δ*gcvP* deletion was to ensure that 5,10-methylene-H_4_F was not derived from glycine cleavage. Indeed, expression of *sdaA*(*C.n*), *fthfl*(*M.t*) and *mthfs*(*M.t*) allowed the Δ*aceA* Δ*gcvP* strain to grow in minimal medium with acetate, glycine, and formate as carbon sources (doubling time of 4.2 h) (Fig. 3b), which suggested that the engineered strain could condense glycine and formate to produce pyruvate and then OAA (marked as blue arrow in Fig. 3a). The controls, without formate addition or *fthfl*(*M.t*) *mthfs*(*M.t*) expression, did not show any growth. Acetate was used as an energy supply and C2 carbon source in this experiment.

Next, we introduced a set of enzymes, including Mtk(*M.c*), Mcl(*M.e*), Agx1(*S.c*), Fthfl(*M.t*), and Mthfs(*M.t*), into the strain Δ*serA* Δ*gcvP*. This strain is expected to synthesize serine only through glycin and formate-derived 5,10-methylene-H_4_F via SHMT (Fig. 2c). The expression of this set of genes allowed the Δ*serA* Δ*gcvP* strain to grow in minimal medium with only pyruvate and formate as carbon sources (doubling time of 2.5 h) (Supplementary Fig. 5D). Similar results could be also observed by rescuing the Δ*serA* Δ*gcvP* strain in minimal medium with supplements of pyruvate and methanol via additional expression of *medh*(*CT4–1*)/*faldh*(*P.p*) (doubling time of 2.1 h) (Fig. 3d). Both results demonstrated the function of part of the modified serine cycle starting from pyruvate to produce serine and acetyl-CoA by assimilation of one formate (or methanol) (marked as blue arrow in Fig. 3c and Supplementary Fig. 5C). The expression of *mtk*(*M.c*) *mcl*(*M.e*) provided the glyoxylate source for glycine synthesis; lacking expression of any necessary genes could not support the strain growth even with C1 compounds supplemented in the medium (Supplementary Fig. 5D and Fig. 3d). The reason for the short doubling time of the Δ*serA* Δ*gcvP* strain growing in the methanol medium is currently unknown. According to above results, we demonstrated a formate (or methanol)-dependent *E. coli* growth by expressing the modified serine cycle genes.

### Demonstration of C1-carbon assimilation in *E. coli*

To further evaluate the capability of the modified serine cycle on C1-carbon assimilation, we performed isotope labeling experiments. An *E. coli* strain *HY106* (Δ*aceB* Δ*glcB* Δ*gcvP* Δ*gcl* Δ*frdB* Δ*ldhA*) was created in order to investigate the effect of expressing the complete cycle genes in vivo (Supplementary Note 1). The gene deletions in *HY106* were to avoid byproduct formation and reactions that could counter part of the serine cycle, and channel the metabolic flux towards acetyl-CoA-derived C2 compounds as the main fermentation products (Supplementary Fig. 6). The required cycle enzymes, including Mtk(*M.c*), Mcl(*M.e*), Agx1(*S.c*), Fthfl(*M.t*), Mthfs(*M.t*), SdaA(*C.n*), GlyA(*E.c*), Medh(CT4–1), and Faldh(*P.p*), were introduced into *HY106* using plasmids *pHY49*, *pHY84*, and *pHY87* (Supplementary Table 4). The *glyA*(*E.c*) was overexpressed in order to increase the cycle flux for methanol condensation with glycine. To enhance the metabolic flow from pyruvate to malate, we additionally overexpressed *pyc* (coding for pyruvate carboxylase)^22^ from *Corynebacterium glutamicum* (Supplementary Fig. 7) and *madh* (coding for malate dehydrogenase) from *E. coli*. After verification of enzyme activities in the engineered strain, 30 mM unlabeled xylose with 200 mM l3C-labeled methanol were used as carbon sources in minimal medium in order to identify the labeling pattern of intracellular metabolites and bioproduct of acetate produced by the strain (Supplementary Note 2 and Supplementary Fig. 8).

**Fig. 4:**
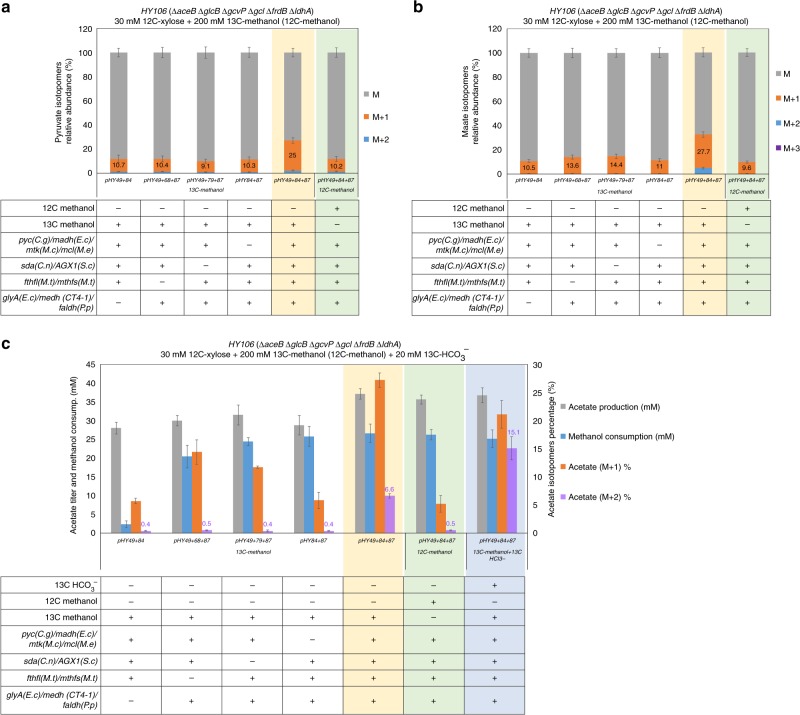
Analysis of the labeling pattern of intracellular metabolites and extracellular acetate produced by the strain *HY106* with expressing different groups of the cycle genes. **a**, **b** Relative abundance of mass isotopomers for intracellular pyruvate (**a**) and malate (**b**) in the engineered strains with expressing different groups of cycle genes. The strains were incubated in minimal medium with supplements of 30 mM xylose and 200 mM 13C-methnaol (or unlabeled methanol) for 3 h. **c** Expression of the modified serine cycle allowed the *E. coli* strain to assimilate methanol and bicarbonate into acetate. The strains were incubated in minimal medium with supplements of 30 mM xylose, 200 mM 13C-methnaol, and 20 mM 13C-HCO_3_^−^ for 20 h. Xylose (30 mM) was consumed after 20 h. Error bars are s.d., *n* = 3

As shown in Fig. 4a, when incubation with 30 mM xylose and 200 mM 13C-methanol in minimal medium for 3 h, the engineered strain that expressed the complete cycle genes showed 25% of intracellular pyruvate labeled with one 13C atom compared to the controls expressing different groups of partial cycle genes. Similar results were also observed in the labeling pattern of intracellular malate. Twenty-eight percent of intracellular malate was labeled with one and 4.5% was labeled by two 13C atoms, respectively, in the engineered strain that expressed the complete cycle genes (yellow part of Fig. 4b). We also applied 30 mM xylose and 200 mM methanol both unlabeled as carbon sources to determine the natural 13 C labeling level of intracellular pyruvate and malate in the strain expressing the complete cycle, which showed lower 13 C labeling percentages of these metabolites under the same conditions (green part of Fig. 4a, b). According to these data, 13C-methanol was indeed assimilated into the cycle metabolites through introducing the modified serine cycle in *E. coli*.

The labeling pattern of acetate produced by the strains was also analyzed. The engineered strain, expressing the complete cycle genes (yellow part of Fig. 4c), produced the highest acetate titer (37 ± 1.4 mM) with clear proportions of M + 1 (about 27.2%, 10 mM) and M + 2 (about 6.6%, 2.4 mM) forms when incubation with 30 mM xylose and 200 mM 13C-methanol in minimal medium for over 20 h. The production of M + 2 acetate demonstrated the capability of the modified serine cycle to condense 13C-methanol into acetyl-CoA in *E. coli* (as predicted in Supplementary Fig. 8E). While the controls, missing expression of *AGX1*(*S.c*) *sdaA*(*C.n*) or *fthfl*(*M.t*) *mthfs*(*M.t*), produced a lower amount of M + 1 (roughly 3.7 mM) and a background level of M + 2 acetate. The M + 1 acetate was detected possibly through assimilation of labeled bicarbonate derived from 13C-methanol oxidation via the partial cycle (as predicted in Supplementary Fig. 8B). Other controls, without expressing *medh*(*CT4–1*) *faldh*(*P.p*) or *mtk*(*M.c*) *mcl*(*M.e*), produced only background levels of both M + 1 and M + 2 acetate in the medium. We also presented the natural labeling percentage of acetate produced from the engineered strain with expressing the complete cycle genes by using both unlabeled xylose and methanol in minimal medium (green part of Fig. 4c).

Next, we incubated the strain, expressing the complete cycle genes, with 30 mM xylose, 200 mM 13C-methanol, and 20 mM 13C-bicarbonate in minimal medium. Compared to 13C-methanol alone (yellow part of Fig. 4c), supplement of both 13C-labeled methanol and bicarbonate did not further increase the acetate titer (36.7 ± 2 mM acetate produced), but it improved the M + 2 acetate proportion (about 15.1%, 5.5 mM) under the same conditions (blue part of Fig. 4c). Thus, these results demonstrated the capability of the modified serine cycle to assimilate both methanol and bicarbonate in vivo.

### Production of acetyl-CoA-derived C2 compounds and ethanol

**Fig. 5:**
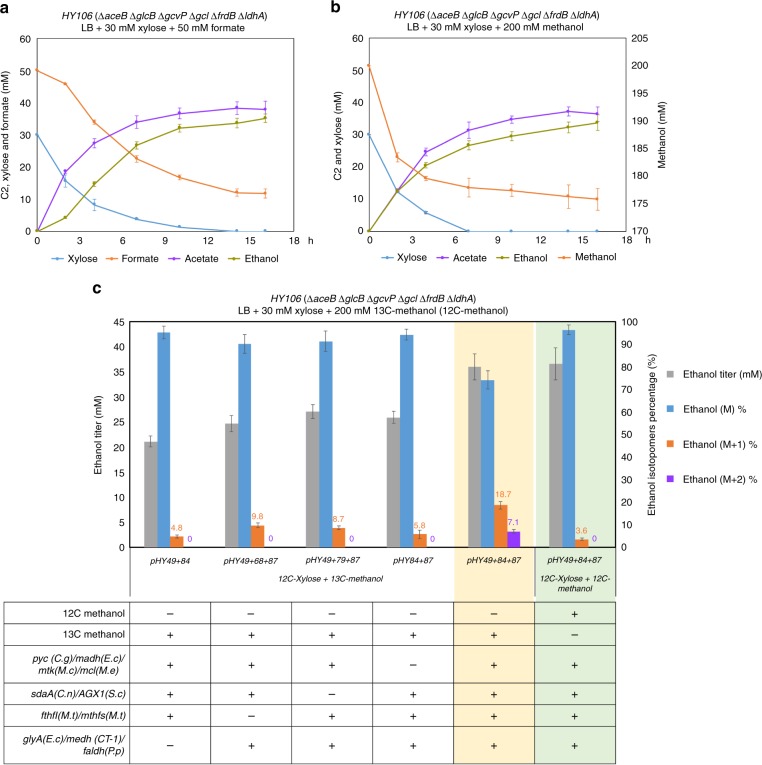
Expression of the modified serine cycle improved the production of C2 compounds and condensed methanol to ethanol in *E. coli*. **a** Time course of the C2 production and carbon consumption during co-assimilation of formate with xylose. The formate assimilation rate was calculated between the second to fourth hour. **b** Time course of the C2 production and carbon consumption during co-assimilation of methanol with xylose. The methanol assimilation rate was calculated between the time zero to second hour. **c** Demonstration of condensing methanol into ethanol in the *E. coli* strain *HY106* with expressing the modified serine cycle genes. The strains were incubated in LB medium with supplements of 30 mM xylose and 200 mM 13C-methnaol (or 200 mM 12C-methnaol) for 20 h. Error bars are s.d., *n* = 3

To investigate the effect of the modified serine cycle on bioproduction, we introduced the complete cycle genes into *HY106* and incubated the engineered strain in Lysogeny Broth (LB) medium with addition of 30 mM xylose and C1 compounds (50 mM formate or 200 mM methanol) under oxygen-limited condition. After 20 h of fermentation, carbon consumption and C2 production were determined. The result showed that expressing the modified serine cycle in *E. coli* increased the production of C2 compounds by co-utilization of C1 compounds with xylose in LB medium (Table 1). Improvement of C2 production was mainly caused by increase in ethanol when comparing the conditions with or without formate (or methanol) supplement (#6 and #7, #10 and #11 in Table 1). Addition of formate resulted in about 32% increase in total C2 titer than the control (#3 and #7 of Table 1). The fastest formate assimilation rate was 0.5 mM/h/OD600 observed between the second to fourth hour (Fig. 5a).

**Table 1:** Construction of the modified serine cycle in *E. coli* improved the production of C2 compounds by co-assimilation of C1 compound with xylose

#	Medium	Xylose consump. (mM)	C1 consump. (mM)	Acetate (mM)	Ethanol (mM)	C1 assimilation rate (mM/h/OD)
**Host:** ***HY106*, No plasmid introduced**.						
1	LB			12 ± 1.8	0	
2	LB + xylose	30		34.1 ± 3.5	22.4 ± 2.9	
3	LB + xylose + formate	30	35.9 ± 1	33.1 ± 0.1	22.4 ± 0.8	
4	LB + xylose + methanol	30	0	35.7 ± 0.1	20 ± 1.1	
**Host:** ***HY106****,* **Plasmids:** ***pHY49*** ***+*** ***84*** **(**Supplementary Table 4**) (expressing the complete serine cycle, but without** ***glyA*(*E.c*))**						
5	LB			10.2 ± 1.6	0.6 ± 0.8	
6	LB + xylose	30		39.6 ± 1.3	20.8 ± 0.5	
7	LB + xylose + formate	30	39 ± 1.1	36.1±2.5	36.9 ± 0.6	0.5
**Host:** ***HY106****,* **plasmids:** ***pHY49*** ***+*** ***84*** ***+*** ***88*** **(**Supplementary Table 4**) (expressing the complete serine cycle, including** ***glyA*(*E.c*))**						
8	LB + xylose + formate	30	39.7 ± 2.1	37.1 ± 1	35.8 ± 2.4	
**Host:** ***HY106****,* **plasmids:** ***pHY49*** ***+*** ***84*** ***+*** ***87*** ***+*** ***89*** **(**Supplementary Table 4**) (expressing the complete serine cycle with** ***glyA*(*E.c*), methanol assimilation genes and ethanol production genes)**						
9	LB			5.1 ± 0.2	0	
10	LB + xylose	30		32.1 ± 1.7	28.5 ± 3.2	
11	LB + xylose + methanol	30	22.6 ± 1.3	34.1 ± 4.4	36.3 ± 2.6	0.7

Since it is not yet possible to condense methanol to ethanol through a one-pot chemical synthesis, thus, it is of interest to investigate whether using the modified serine cycle could perform such reaction in *E. coli*. To do so, we additionally introduced Pdup (CoA-acylating aldehyde dehydrogenases) from *Salmonella enterica* and AdhB (alcohol dehydrogenase 2) from *Zymomonas mobilis* into *HY106* to facilitate ethanol production (Supplementary Fig. 6). Addition of methanol in LB medium increased ethanol titer by approximately 62% in the engineered strain with expressing the complete cycle genes compared to the control (#2 and #11 of Table 1). The fastest methanol assimilation rate was 0.7 mM/h/OD600 (Fig. 5b and Supplementary Fig. 9) and the consumption of xylose and methanol was at about 1:0.7 molar ratio in LB medium. Adding 13C-methanol and unlabeled xylose in the culture, we identified the production of ethanol with M + 1 (18.7%, about 6.7 mM) and M + 2 (7.1%, about 2.6 mM) forms in the engineered strain expressing the complete cycle genes (yellow part of Fig. 5c). While the controls, whether expressing different groups of partial cycle genes or expressing the complete cycle genes but supplied with unlabeled methanol in LB medium (green part of Fig. 5c), both displayed lower ethanol titer in M + 1 and no production in M + 2 form (Supplementary Fig. 10 and Supplementary Table 5). Thus, according to these results, we demonstrated the first microbial condensation of methanol to ethanol in *E. coli*.

## Discussion

Chemical approaches to build C–C bounds from C1 compounds are challenging and require high capital expenditure. Therefore, utilization of an industrially relevant microorganism, such as *E. coli*, to assimilate various C1 compounds is of interest, not only to broaden the substrate range for microbes, but also to achieve reactions currently difficult for chemical processes. For example, methanol condensation to ethanol by use of one-pot chemical synthesis has not been reported, except by the synthetic MCC^8^ using purified enzymes in vitro. Engineering methylotrophs to produce various chemicals is feasible but time-consuming, since the genetic tools in those microorganisms are not as efficient as in *E. coli*.

Here we designed and constructed a modified serine cycle for *E. coli* to assimilate various groups of C1 compounds. We demonstrated its capability of assimilating C1 compounds in engineered strains by growth complementation and isotope labeling experiments. Construction of the modified serine cycle in *E. coli* improved the production of C2 compounds by co-assimilation of C1 compounds with xylose. We further demonstrated methanol condensation to ethanol using this engineered strain.

Compared to the natural serine cycle, the modified serine cycle simplifies the enzymatic steps for methanol oxidation to formate, and utilizes alanine-glyoxylate transaminase (Agt) to convert glyoxylate to glycine by using alanine as an amino donor in order to bypass the deleterious effect of hydroxypyruvate reductase in *E. coli*. Recent paper^20^ reported to use a threonine biosynthesis and cleavage route to generate glycine for formate assimilation. In contrast to Mtk/Mcl/Agx1 used in the modified serine cycle, this threonine metabolic route includes more enzymatic steps and consumes one more ATP in producing glycine from OAA (Supplementary Fig. 11).

The modified serine cycle utilizes a simple cyclic network to assimilate various C1 compounds simultaneously, but requires 3 ATP consumption to produce each acetyl-CoA (Supplementary Table 3). The modified serine cycle can also support pyruvate synthesis by assimilation of two formate (or methanol) with one bicarbonate (Supplementary Fig. 3), which can ultimately enable *E. coli* strain to grow on a reduced C1 compound alone.

## Methods

### Strain construction

All strains used in this study are listed in Supplementary Table 4. Gene deletions were performed by P1 transduction with a single knockout strain from Keio Collection except the strain Δ*serA* Δ*gcvP*. The *serA* and *gcvp* are close to each other in genome, we used lambda-red recombination system to replace *gcvp* with a chloramphenicol resistant gene (*cat*) in the Δ*serA* strain. The gene knockouts were verified by PCR with primers flanking the deleted locus.

### Plasmid construction

All plasmids used in this study are listed in Supplementary Table 4. The plasmids were constructed by Gibson DNA assembly^23^. The primers used for the cloning are shown in Supplementary Table 6.

### In vitro enzyme assay

*The Medh assay*: Medh catalyzes the oxidation of methanol to formaldehyde with the formation of NADH, which can be recorded at 340 nm. The reaction was set up at 37 °C with a final volume of 200 μL containing 100 mM NaHCO_3_ buffer (pH 9.5), 5 mM MgCl_2_, 500 mM methanol, 2 mM NAD, 20 μg Medh purified protein.

*The Pyc-Madh assay*: Pyc can catalyze the reaction of carboxylation of pyruvate to form OAA. Madh reduces OAA to generate malate with the oxidation of NADH, which can be recorded at 340 nm. The reaction was set up at 37 °C with a final volume of 200 μL containing 50 mM Tris-Cl pH 7.5, 5 mM MgCl_2_, 5 mM pyruvate, 5 mM NaHCO_3_, 2.5 mM ATP, 0.25 mM NADH, 50 μM acetyl-CoA, 1 μL Madh (acquired from Sigma), 10 μg Pyc purified protein.

### Protein synthesis and purification

The gene was fused with a His-tag at the N-terminal and cloned under T7 promoter. The plasmid was transformed into *E. coli BL21* (*DE3*) for protein synthesis. Overnight culture was inoculated (2% vol/vol) into fresh LB medium. Culture was grown at 37 °C with 250 rpm agitation to mid-log phase (OD of 0.4–0.6), and induced with 0.1 mM IPTG (Isopropyl β-d-1-thiogalactopyranoside; Zymo Research) for additional 6 h at 30 °C. Cell pellet was lysed with 0.1 mm diameter glass beads at 4 °C, and protein was purified by His-Spin Protein Mini-prep columns (Zymo Research). Concentration of purified protein was measured using BioRad Protein Assay kit, and protein purity was verified by standard SDS-PAGE with Coomassie staining.

### Growth rescue of *E. coli* strains

Overnight culture was inoculated (2% vol/vol) in fresh LB medium and grown until the mid-log phase. IPTG (0.1 mM) was added into culture to induce protein synthesis at 30 °C for 6 h. One milliliter of culture was harvested and washed three times with equal volume of minimal medium. Sixty microliters of the culture were inoculated (2% vol/vol) into minimal medium (3 mL) for growth test. Minimal medium contained M9 salts (12.8 g/L Na_2_HPO_4_·7H_2_O, 3 g/L KH_2_PO_4_, 0.5 g/L NaCl, 1 g/L NH_4_Cl), 1 mM MgSO_4_, 0.1 mM CaCl_2_, 0.1 mM ammonium iron(II) sulfate, 0.1 mg/mL thiamine hydrochloride, 0.1 mM IPTG with appropriate antibiotics. The medium was then supplemented with carbon sources (all from Sigma-Aldrich) as noted in the study.

### The C2 production

Overnight culture was inoculated (2% vol/vol) in fresh LB medium and grown to the mid-log phase. IPTG (0.2 mM) and 20 mM xylose were added into culture for protein synthesis at 30 °C for 6 h. Ten milliliters of culture were harvested and re-suspended into 2 mL fresh LB medium supplemented with 0.1 mM IPTG, 30 mM xylose, and C1 compounds (50 mM formate or 200 mM methanol) in a glass tube (BD vacutainer glass tube). Supernatant of culture was diluted for five-fold and filtered by Amicon 10 kDa protein filters. Twenty microliters of sample were applied to HPLC (high-performance liquid chromatography) with a Bio-Rad Aminex HPX87 column (30 mM H_2_SO_4_; 0.4 mL/min; column temperature 30 °C). Organic acids were detected using a multiple wavelength detector at 210 nm, and xylose and methanol were measured by a refractive index detector. Ethanol was determined by GC-FID (flame ionization detector) (Agilent Technologies). 1-Propanol was used as the internal standard.

### Isotope labeling experiments on intracellular metabolites

Overnight culture was inoculated (2% vol/vol) in fresh LB medium and grown to the mid-log phase. IPTG (0.2 mM) and 20 mM xylose were added to culture for protein synthesis at 30 °C for 6 h. Ten milliliters of culture were harvested and re-suspended into 2 mL minimal medium supplemented with 0.1 mM IPTG, 30 mM xylose, and 200 mM 13C-methanol in a glass tube (BD vacutainer glass tube). For determination of intracellular pyruvate, the tubes were shaken in a 37 °C incubator for 3 h. Four milliliters of culture (from two tubes) were spin down, and total pellets were collected and re-suspended in 0.4 mL buffer (5% formic acid in 100 mM MOPS (pH 7.0)). Then pellet was lysed with 0.1 mm diameter glass beads and spin at maximal speed for 10 min at 4 °C. Two hundred and seventy microliters of supernatant were mixed with 30 μL of 100 mM phenylhydazine at room temperature for 10 min to derivatization of pyruvate to form pyruvate-phenylhydrazone^24^. For determination of intracellular malate, four milliliters of culture (from two tubes) were spin down, and total pellets were re-suspended in 0.4 mL buffer (methanol:water (75:25, v/v)). Then pellet was lysed with 0.1 mm diameter glass beads and spin at maximal speed for 10 min at 4 °C. Derivatization of malate was as described in Han et al.^25^. Briefly, 100 μL of supernatant were mixed with 50 μL of 250 mM 3-nitrophenylhydrazine in 50% methanol, 50 μL of 150 mM carbodiimide in methanol, and 50 μL of 7.5% pyridine in 75% methanol. The mixture was incubated at 30 °C for 30 min. The supernatant was filtered by Amicon 10 kDa protein filters (EMD-Amicon). The LC-MS (liquid chromatography-mass spectrometry) analyses were performed on a Shimadzu 2020 EVLC-MS (Phenomenex kinetex, 1.7 µm, 2.0 × 100 mm, C18 column) using positive and negative mode electrospray ionization with a linear gradient of 5–95% acetonitrile–H_2_O supplemented with 0.1% (v/v) formic acid in 15 min followed by 95% acetonitrile for 5 min with a flow rate of 0.3 mL/min.

### Isotope labeling experiments on C2 compounds

Overnight culture was inoculated (2% vol/vol) in fresh LB medium and grown to the mid-log phase. IPTG (0.2 mM) and 20 mM xylose were added to culture for protein synthesis at 30 °C for 6 h. Ten milliliters of culture were harvested and re-suspended into 2 mL minimal medium supplemented with 0.1 mM IPTG, 30 mM xylose, 200 mM 13C-methanol, and 20 mM 13C-bicarbonate. The culture was shaken in a 37 °C incubator for 20 h. Two milliliters of culture were spin down at maximal speed, and supernatant was diluted for five-fold and filtered by Amicon 10 kDa protein filters. Twenty microliters of sample were applied to HPLC with a Bio-Rad Aminex HPX87 column to measure the acetate titer. 13C-labeled acetate was determined by GC-MS as described in Lin et al.^26^. The oven temperature was started at 70 °C and held for 1 min, followed by a ramp at 20 °C/min and a 2-min hold at 240 °C.

### Data analysis

Data are presented as mean ± s.d. (standard deviation) unless otherwise indicated in figure legends. For growth and production assays, three biological replicates of each strain were tested.

## Electronic supplementary material

Supplementary Information

## Data Availability

All the genes used are listed in Supplementary Table 7. Their sequences can be obtained by searching accession ID and the associated organism in Biocyc (https://biocyc.org/). All other relevant data are available from the authors upon request.

## References

[1] Sawatdeenarunat C (2016). Anaerobic biorefinery: current status, challenges, and opportunities. Bioresour. Technol..

[2] Caballero A, Pérez PJ (2013). Methane as raw material in synthetic chemistry: the final frontier. Chem. Soc. Rev..

[3] Li H (2012). Integrated electromicrobial conversion of CO_2_ to higher alcohols. Science.

[4] Jönsson LJ, Martín C (2016). Pretreatment of lignocellulose: formation of inhibitory by-products and strategies for minimizing their effects. Bioresour. Technol..

[5] Kozlowski JT, Davis RJ (2013). Heterogeneous catalysts for the Guerbet coupling of alcohols. ACS Catal..

[6] Smejkalová H, Erb TJ, Fuchs G (2010). Methanol assimilation in *Methylobacterium extorquens* AM1: demonstration of all enzymes and their regulation. PLoS ONE.

[7] Whitaker WB (2017). Engineering the biological conversion of methanol to specialty chemicals in *Escherichia coli*. Metab. Eng..

[8] Meyer F (2018). Methanol-essential growth of *Escherichia coli*. Nat. Commun..

[9] Woolston BM, King JR, Reiter M, Van Hove B, Stephanopoulos G (2018). Improving formaldehyde consumption drives methanol assimilation in engineered *E. coli*. Nat. Commun..

[10] Bogorad IW (2014). Building carbon-carbon bonds using a biocatalytic methanol condensation cycle. Proc. Natl. Acad. Sci. USA.

[11] Crowther GJ, Kosály G, Lidstrom ME (2008). Formate as the main branch point for methylotrophic metabolism in *Methylobacterium extorquens AM1*. J. Bacteriol..

[12] Kalyuzhnaya MG, Lidstrom ME (2005). QscR-mediated transcriptional activation of serine cycle genes in *Methylobacterium extorquens AM1*. J. Bacteriol..

[13] Ravnikar PD, Somerville RL (1987). Genetic characterization of a highly efficient alternate pathway of serine biosynthesis in *Escherichia coli*. J. Bacteriol..

[14] Huo YX (2011). Conversion of proteins into biofuels by engineering nitrogen flux. Nat. Biotechnol..

[15] Nuñez MF, Pellicer MT, Badia J, Aguilar J, Baldoma L (2001). Biochemical characterization of the 2-ketoacid reductases encoded by *ycdW* and *yiaE* genes in *Escherichia coli*. Biochem. J..

[16] Mainguet SE, Gronenberg LS, Wong SS, Liao JC (2013). A reverse glyoxylate shunt to build a non-native route from C4 to C2 in *Escherichia coli*. Metab. Eng..

[17] Giffin MM, Modesti L, Raab RW, Wayne LG, Sohaskey CD (2012). *ald* of *Mycobacterium tuberculosis* encodes both the alanine dehydrogenase and the putative glycine dehydrogenase. J. Bacteriol..

[18] Grabowski R, Hofmeister AE, Buckel W (1993). Bacterial L-serine dehydratases: a new family of enzymes containing iron-sulfur clusters. Trends Biochem. Sci..

[19] Okamura-Ikeda K, Ohmura Y, Fujiwara K, Motokawa Y (1993). Cloning and nucleotide sequence of the *gcv* operon encoding the *Escherichia coli* glycine-cleavage system. Eur. J. Biochem..

[20] Yishai O, Goldbach L, Tenenboim H, Lindner SN, Bar-Even A (2017). Engineered assimilation of exogenous and endogenous formate in *Escherichia coli*. ACS Synth. Biol..

[21] Wu TY (2016). Characterization and evolution of an activator-independent methanol dehydrogenase from *Cupriavidus necator N-1*. Appl. Microbiol. Biotechnol..

[22] Peters-Wendisch PG (1998). Pyruvate carboxylase from *Corynebacterium glutamicum*: characterization, expression and inactivation of the *pyc* gene. Microbiology.

[23] Gibson DG (2009). Enzymatic assembly of DNA molecules up to several hundred kilobases. Nat. Methods.

[24] Petrarulo Michele (1998). High-performance liquid chromatographic determination of glyoxylic acid in urine. J. Chromatogr. B Biomed. Sci. Appl..

[25] Han J, Gagnon S, Eckle T, Borchers CH (2013). Metabolomic analysis of key central carbon metabolism carboxylic acids as their 3-nitrophenylhydrazones by UPLC/ESI-MS. Electrophoresis.

[26] Lin PP (2018). Construction and evolution of an Escherichia coli strain relying on nonoxidative glycolysis for sugar catabolism. Proc. Natl. Acad. Sci. USA.

