# Supplementary material for: A modified serine cycle in Escherichia coli coverts methanol and CO2 to two-carbon compounds
Source: Nat Commun. 2018 Sep 28;9:3992. doi: 10.1038/s41467-018-06496-4 (PMC6162302; doi:10.1038/s41467-018-06496-4)
Supplement: Supplementary file 1 — Supplementary Information [file 41467_2018_6496_MOESM1_ESM.pdf]

A modified serine cycle in *Escherichia coli* coverts methanol and CO_2 to two-carbon compounds

Yu *et al*

Supplementary Note 1

Construction of the *HY106* strain to evaluate the effect of expressing the complete cycle genes *in vivo*

To investigate the effect of the modified serine cycle on C1-carbon assimilation, we first expressed the complete required cycle genes, including *mtk*(*M.c*), *mcl*(*M.e*), *AGX1*(*S.c*), *sdaA*(*C.n*), *fthfl*(*M.t*) and *mthfs*(*M.t*), in the strain Δ*serA* Δ*gcvP*. However, this *E. coli* strain grew much poorly compared to the previous result under the same condition (Supplementary Fig. 4D), which might be caused by shortage of serine because of the *serA* deletion and the SdaA(*C.n*)-mediated serine deamination. Thus, an *E. coli* strain *HY106* (Δ*aceB* Δ*glcB* Δ*gcvP* Δ*gcl* Δ*frdB* Δ*ldhA*) was created (Supplementary Fig. 6), which did not show auxotrophic growth defect and was used to investigate the effect of expressing the complete cycle genes. The gene deletions in *HY106* were to avoid byproduct formation and reactions that could counter part of the modified serine cycle, and further channel the metabolic flux towards acetyl-CoA derived C2 compounds (acetate and ethanol) as the main fermentation products[1]. AceB(*E.c*) and GlcB(*E.c*)[2] are deleted since they act as malate synthases that catalyze the reverse reaction of Mtk/Mcl. Gcl(*E.c*)[3] catalyzes the glyoxylate condensation to form tartronate semialdehyde. GcvP(*E.c*) performs the glycine decarboxylation. LdhA and FrdABCD are lactate dehydrogenase and fumarate reductase, specific for production of D-lactate and succinate, respectively.

Supplementary Note 2

Prediction of the acetate labeling pattern produced by the modified serine cycle in *E. coli*

In the engineered *E. coli* strain, methanol can be oxidized to formate by expressing heterologous enzymes Medh(CT4-1)/Faldh(*P.p*). The intermediate formate is further converted to 5,10-methylene-H_4F catalyzed by Fthfl(*M.t*)/Mthfs(*M.t*), or to CO_2 equivalent by endogenous formate dehydrogenases Fdo(*E.c*) and Fdn(*E.c*). As the native pathway produces acetyl-CoA through pyruvate decarboxylation (Supplementary Fig. 8A), *E. coli* strain can only incorporate 13C labeled methanol into acetyl-CoA by

constructing the modified serine cycle, resulting in two forms of acetyl-CoA (M+1 and M+2) from three possibilities. The first possibility is unlabeled pyruvate assimilates 13C labeled bicarbonate, derived from oxidation of 13C-methanol, to generate oxaloacetate and further split into single-carbon labeled acetyl-CoA (M+1) and unlabeled glyoxylate (Supplementary Fig. 8B). In the first possibility, acetyl-CoA (M+1) is produced only through the partial cycle, while the remaining possibilities may produce 13C labeled acetyl-CoA from the complete cycle. The unlabeled glyoxylate (produced via Supplementary Fig. 8B and 8C) assimilates labeled 5,10-methylene-H_4F, derived from 13C-methanol, to produce single-carbon labeled serine. The M+1 serine is converted to pyruvate, and then carboxylated with either unlabeled bicarbonate (Supplementary Fig. 8D) or labeled bicarbonate (Supplementary Fig. 8E) to produce M+1 or M+2 form of oxaloacetate, respectively. The labeled malate can be split into unlabeled glyoxylate and single (or double)-carbon labeled acetyl-CoA (Supplementary Fig. 8D and 8E).

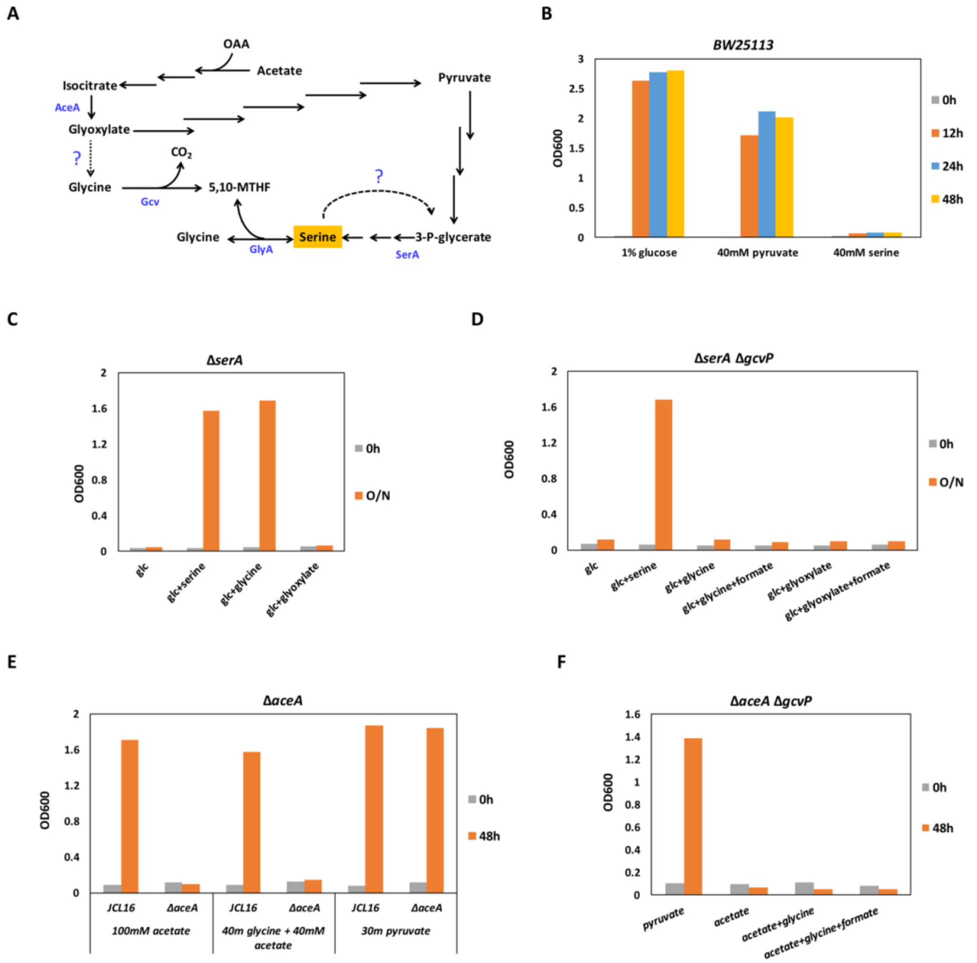

Supplementary Figure 1. Serine and glycine metabolism in *E. coli*.

(A) In *E. coli*, serine is synthesized either from two glycine or one 3-P-glycerate. The C1-carbon carrier 5,10-methylene-H_4F can be produced by glycine-cleavage or glycine synthesis from serine. The questionmarks indicate that *E. coli* wt strain doesn't have such enzymatic activities under normal growth condition, which are further demonstrated in (B) and (C). Gcv: glycine cleavage complex; GlyA: serine hydroxymethyltransferase (SHMT); SerA: D-3-phosphoglycerate dehydrogenase. AceA: isocitrate lyase.
(B) The wt strain *BW25113* cannot grow in minimal medium with serine as the sole carbon source.
(C) The *serA* deletion causes *E. coli* strain to be a serine auxotroph, which can be rescued by supplement of serine or glycine in glucose minimal medium. However, addition of glyoxylate cannot rescue the growth of the Δ*serA* strain, suggesting that conversion of glyoxylate to glycine is inefficient. O/N: overnight, 0 h: time zero.
(D) The Δ*serA* Δ*gcvP* strain can grow in glucose minimal medium with only serine addition, but not glycine or glyoxylate addition, compared to the single *serA* deletion (C). This result indicates the role of GcvP in the serine synthesis.
(E) AceA(*E.c*) acts as an isocitrate lyase, which catalyzes the key step of the glyoxylate cycle. Its deletion causes *E. coli* strain cannot grow in minimal medium with acetate (C2) as the sole carbon source, but can grow on pyruvate (C3) or succinate (C4).
(F) The Δ*aceA* Δ*gcvP* strain cannot grow in minimal medium by using acetate/glycine or acetate/glycine/formate as carbon sources.

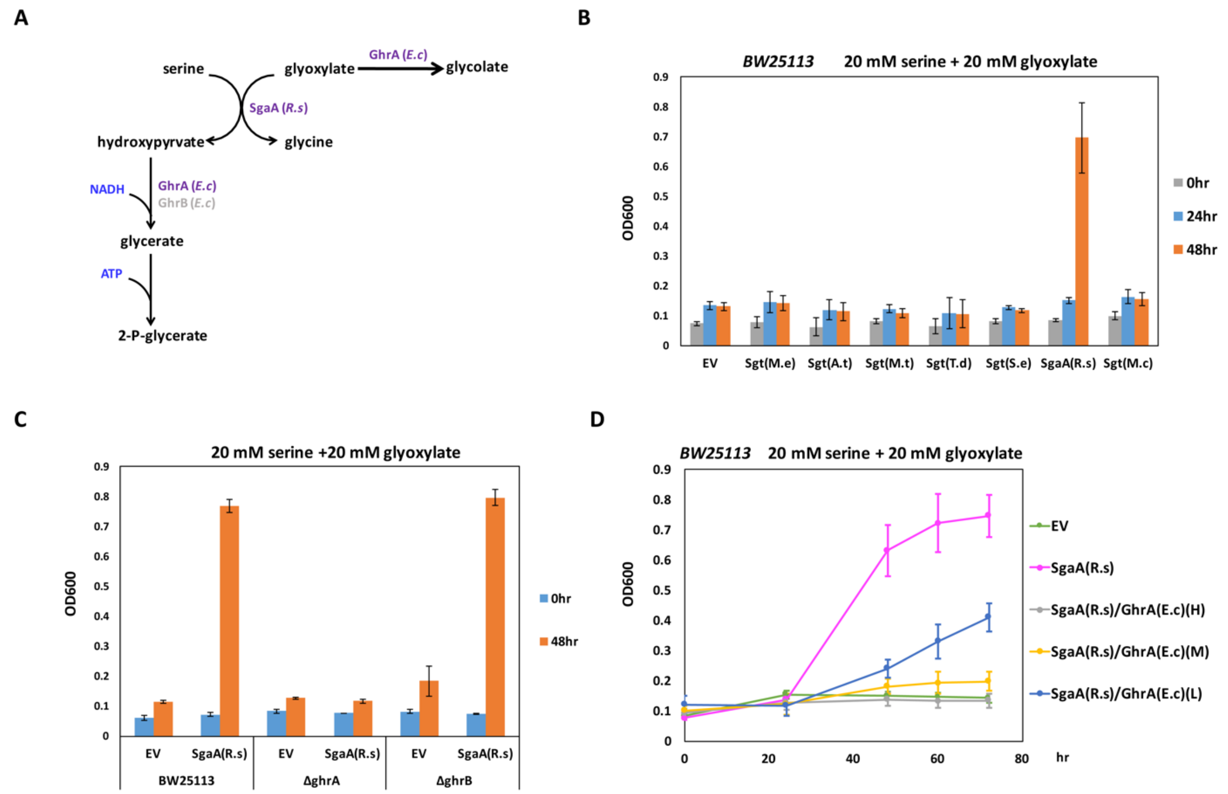

Supplementary Figure 2. Investigation of the GhrA function in *E. coli*.
(A) Pathway from serine and glyoxylate to produce 2-phosphoglcerate for *E. coli* growth. SgaA: serine-glyoxylate transaminase. GhrA, GhrB: glyoxylate (hydroxypyruvate) reductase.
(B) Expression of *sgaA* (coding for serine-glyoxylate transaminase) from *Rhodobacter sphaeroides* allowed *E. coli* wt strain to grow in minimal medium with serine and glyoxylate as carbon sources. It suggested that SgaA(*R.s*) was functional in converting serine and glyoxylate to produce hydroxypyruvate and glycine, hydroxypyruvate could be further reduced to glycerate by endogenous hydroxypyruvate reductase to support growth, as shown in (A).
M.e: *Methylobacterium extorquens*. *A.t*: *Arabidopsis thaliana*. *M.t*: *Moorella thermoacetic*. *T.d*: *Treponema denticola*. *S.e*: *Synechococcus elongatus PCC 7942*. *R.s*: *Rhodobacter sphaeroides*. *M.c*: *Methylococcus capsulatus* (*Bath*).
(C) Deletion of *ghrA*, but not *ghrB*, abolished the phenotype observed in (B), suggesting that GhrA was the major hydroxypyruvate reductase in *E. coli*.
(D) Increased expression levels of *ghrA*(*E.c*) gradually slowed down the growth rate of *E. coli* wt in minimal medium with serine and glyoxylate as carbon sources. The expression level of *ghrA*(*E.c*) was controlled by synthetic ribosome binding site (RBS), designed by the RBS calculator (https://salislab.net/software/). GhrA(*E.c*)(H), GhrA(*E.c*)(M) and GhrA(*E.c*)(L) represented high, medium and low expression levels of *grhA*(*E.c*). Each RBS was designed with 10-fold difference on the translation rate. The RBS sequences were listed in Supplementary Table 6. EV means empty vector.
Error bars are s.d., n=3.

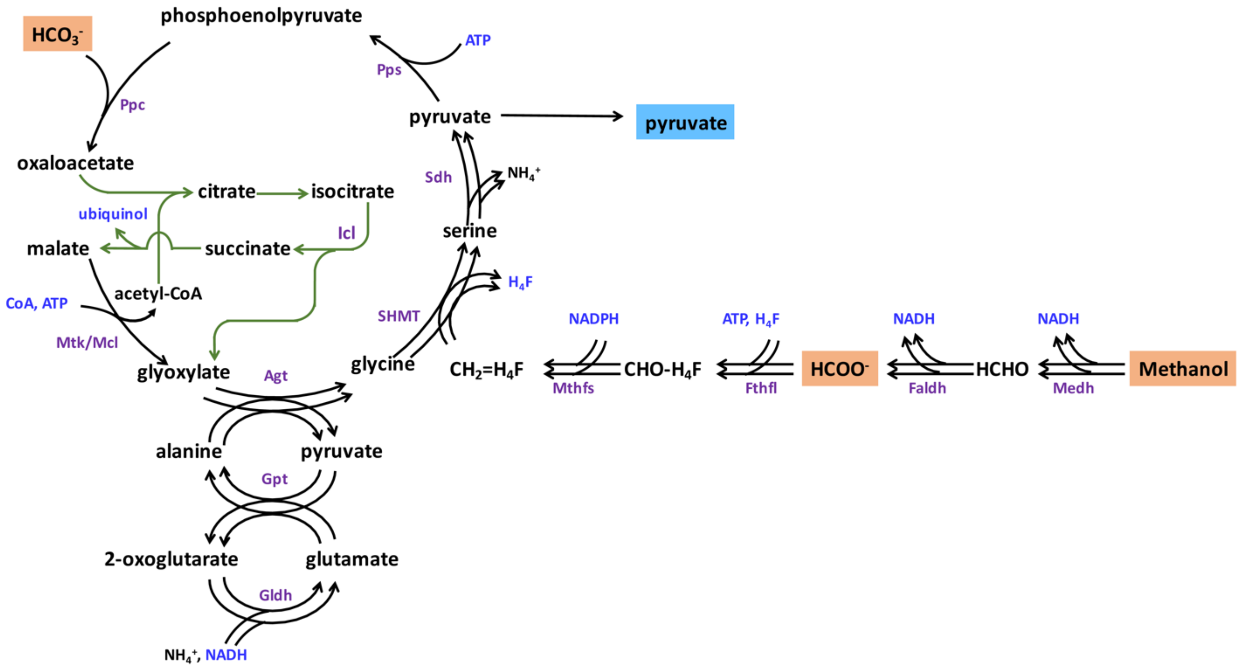

Supplementary Figure 3. Illustration of the modified serine cycle for pyruvate synthesis. Compared to Fig. 1B, this form of the modified serine cycle integrates with the partial glyoxylate cycle (labeled as green lines), which allows the pathway to assimilate two C1-carbon units, derived from formic acid (or methanol), and one bicarbonate to produce one pyruvate. Icl: isocitrate lyase.

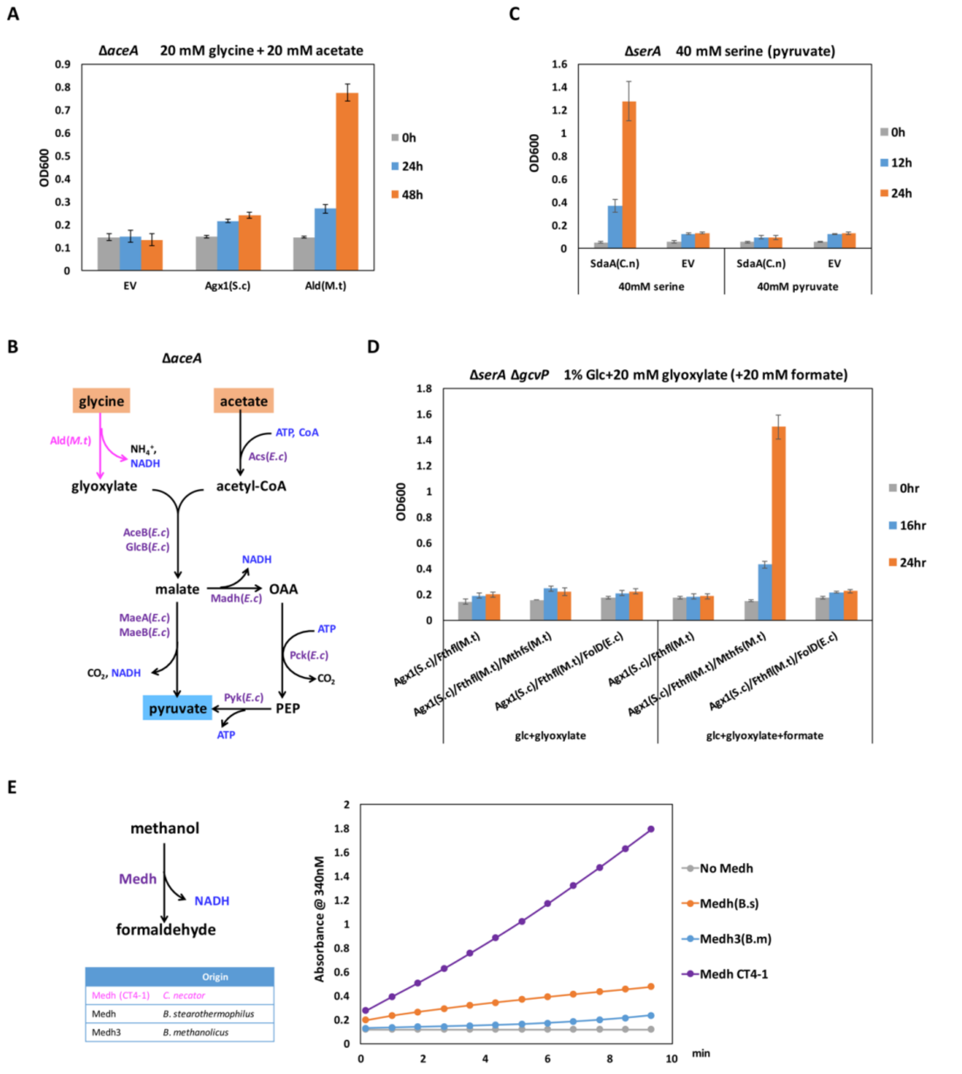

Supplementary Figure 4. Investigation of suitable enzymes for the modified serine cycle.

(A,B) Expression of *ald*(*M.t*) allowed the Δ*aceA* strain to grow in minimal medium with acetate and glycine as carbon sources (A). Ald(*M.t*) catalyzed the reaction to convert glycine to glyoxylate, which could be condensed with acetyl-CoA to generate malate for growth-supporting, as shown in (B). Enzymes labeled in purple are endogenous enzymes in *E. coli*. Together with the result of Fig. 2A, it suggested that the function of Ald(*M.t*) preferred to catalyze the reaction of converting glycine to glyoxylate in *E. coli*. Acs: acetyl-CoA synthase. AceB, GlcB: malate synthase. MaeA, MaeB: malate dehydrogenase. Pck: phosphoenolpyruvate carboxykinase. Pyk: pyruvate kinase.

(C) Expression of *sdaA*(*C.n*) could support the *ΔserA* strain to grow in minimal medium with serine addition, but not pyruvate, which indicated that SdaA(*C.n*) catalyzed the serine deamination irreversibly *in vivo*.

(D) Comparison of the activity between Fthfl(*M.t*)/Mthfs(*M.t*) and Fthfl(*M.e*)/FolD(*E.c*). The results showed that Fthfl(*M.t*)/Mthfs(*M.t*) displayed higher activities to rescue the growth defect of the Δ*serA* Δ*gcvP* strain in glucose minimal medium with glyoxylate/formate supplements.

(E) *In vitro* enzymatic assay showed that Medh(CT4-1) displayed the highest enzymatic activity on methanol oxidation among the enzymes tested at 37°C. NADH formation was recorded at 340 nm. 20 ug of each purified protein was used.

EV means empty vector. Error bars are s.d., n=3.

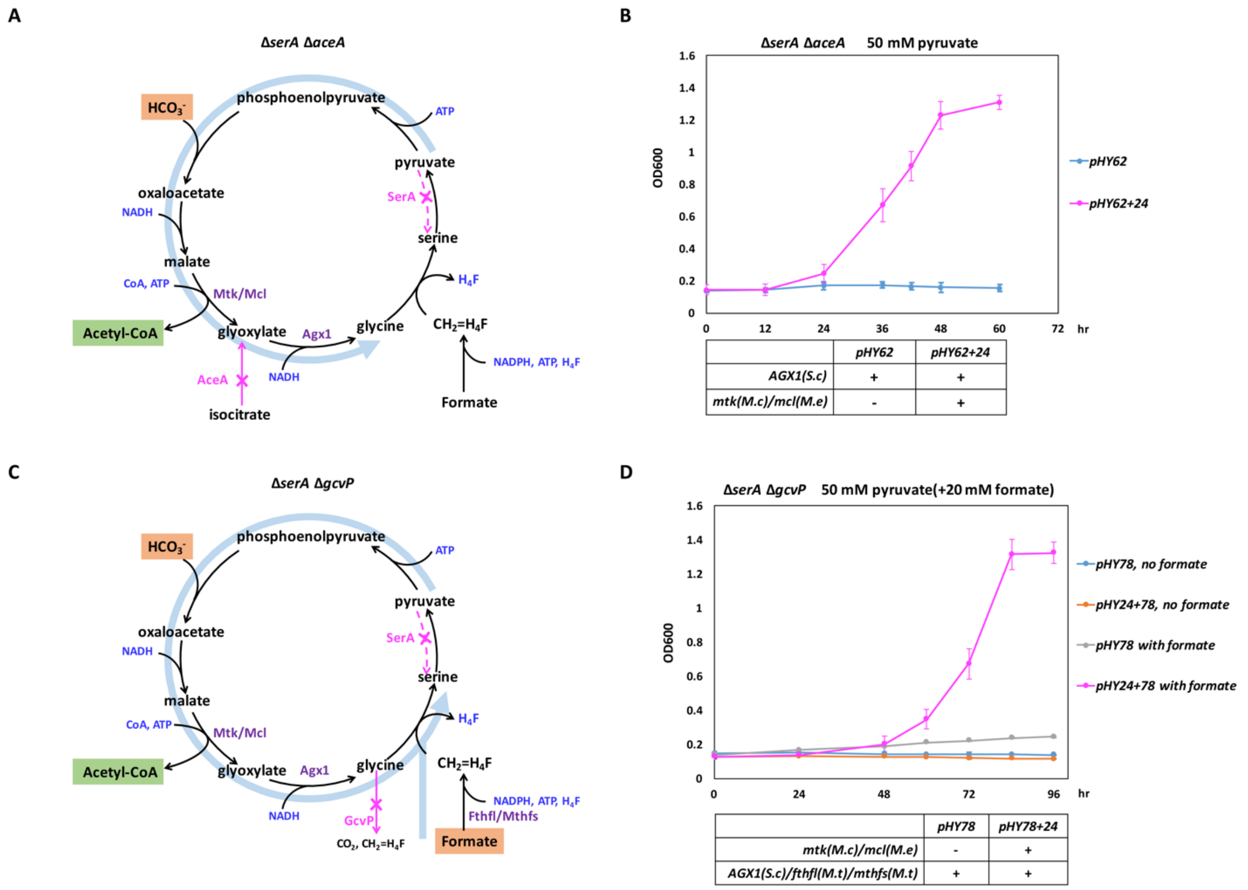

Supplementary Figure 5. Construction of the modified serine subpathway in *E. coli*.

(A,B) Introduction of Mtk(*M.c*), Mcl(*M.e*) and Agx1(*S.c*) allowed the Δ*serA* Δ*aceA* strain to grow in minimal medium with 50 mM pyruvate as the sole carbon source (B), however, expression of *AGX1*(*S.c*) alone did not show similar effect under the same conditions, which demonstrated the function of the modified serine subpathway marked as blue arrow in (A).

(C,D) Expression of a set of heterologous genes, including *mtk*(*M.c*), *mcl*(*M.e*), *AGX1*(*S.c*), *fthfl*(*M.t*) and *mthfs*(*M.t*), allowed the Δ*serA* Δ*gcvp* strain to grow in minimal medium with pyruvate and formate as carbon sources (D), while the controls without formate addition or omitting *mtk*/*mcl* expression did not show obvious growth, which indicated the function of the modified serine subpathway marked as blue arrow in (C).

Error bars are s.d., n=3.

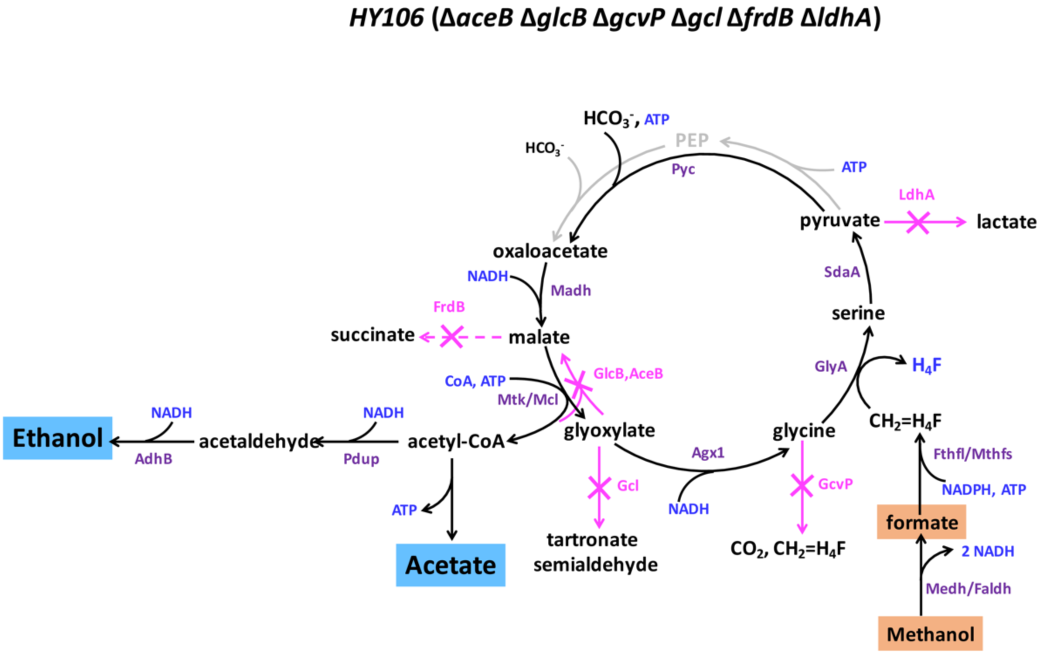

Supplementary Figure 6. Rationale of using the *E. coli* strain *HY106* (Δ*aceB* Δ*glcB* Δ*gcvp* Δ*gcl* Δ*frdB* Δ*ldhA*) to investigate the effect of expressing the modified serine cycle *in vivo* (Supplementary Note 1). The genes *pyc*(*C.g*) and *madh*(*E.c*) were overexpressed in order to enhance the carbon flux from pyruvate to malate, *pdup*(*S.e*) and *adhB*(*Z.m*) were expressed to facilitate ethanol production.

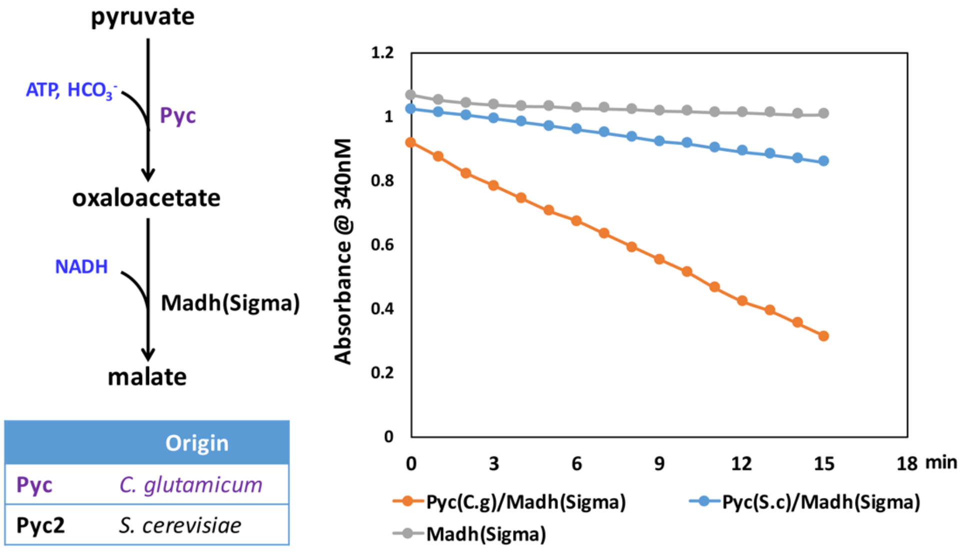

Supplementary Figure 7. Determination of the activity of Pyc(*C.g*) *in vitro*. Pyc(*C.g*) displayed much higher enzymatic activity than the one from *S. cerevisiae*. NADH consumption was recorded at 340 nm. 10 ug of each purified protein was used. Malate dehydrogenase (Mdh) was acquired from Sigma.

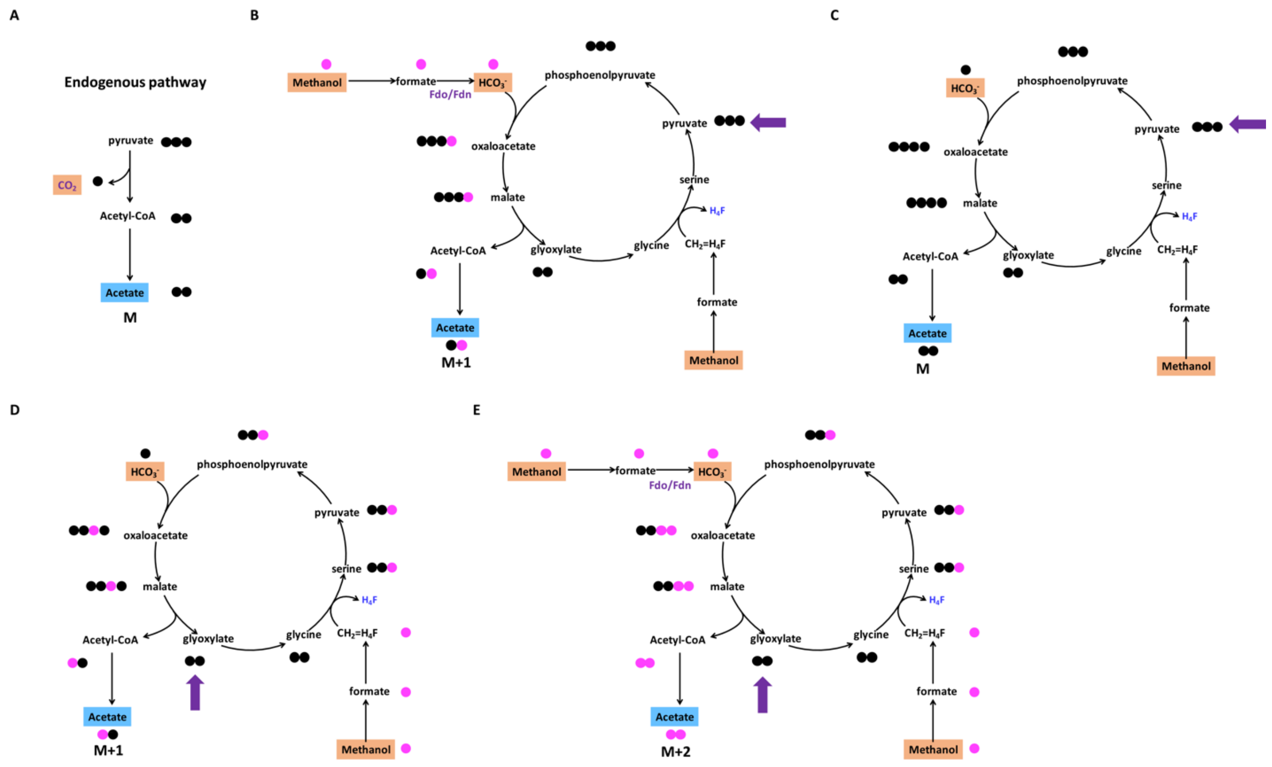

Supplementary Figure 8. Prediction of the labeling pattern of acetate produced by the engineered *E. coli* strain with expressing the modified serine cycle genes. Magenta dot stands for 13C labeled methanol or its derived C1-carbon. Black dot indicates unlabeled carbon. Purple arrow means the starting carbon source.

(A) Acetyl-CoA can be produced via pyruvate decarboxylation through endogenous pyruvate dehydrogenase complex.
(B) Unlabeled pyruvate assimilates 13C labeled bicarbonate, derived from oxidation of 13C-methanol, to generate labeled malate, and further splits into single-carbon labeled acetyl-CoA (M+1) and unlabeled glyoxylate.
(C) Unlabeled pyruvate assimilates unlabeled bicarbonate to generate malate, and splits into both unlabeled acetyl-CoA and glyoxylate.
(D) Unlabeled glyoxylate (produced from B and C) assimilates labeled 5,10-methylene-H_4F, derived from 13C-methanol, to produce single-carbon labeled serine. The M+1 serine is converted to pyruvate and assimilates unlabeled bicarbonate to produce M+1 form of OAA. The labeled malate can be split into single-carbon labeled acetyl-CoA (M+1) and unlabeled glyoxylate.
(E) Unlabeled glyoxylate (produced from B and C) assimilates labeled 5,10-methylene-H_4F, derived from 13C-methanol, to produce single-carbon labeled serine. The M+1 pyruvate assimilates 13C labeled bicarbonate, derived from oxidation of 13C-methanol, to produce M+2 OAA. The labeled malate can be split into double-carbon labeled acetyl-CoA (M+2) and unlabeled glyoxylate.

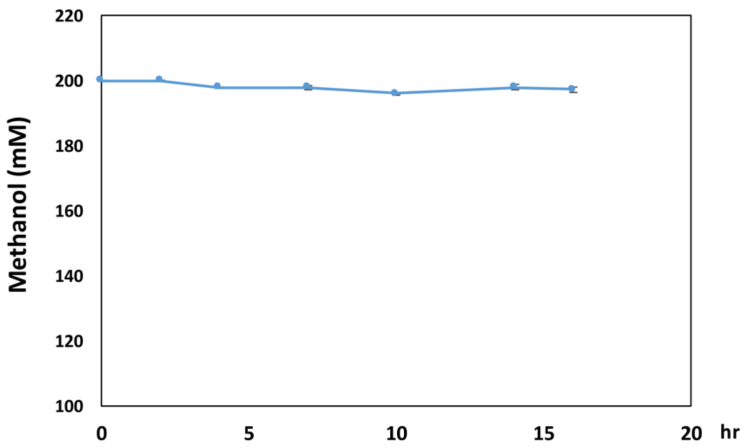

Supplementary Figure 9. Determination of the methanol evaporation control. To minimize the methanol evaporation at 37°C shaker, we performed the bioproduction experiments under oxygen-limited condition in a sealed tube (BD vacutainer glass tube). The methanol evaporation control was determined in LB medium with xylose/methanol addition at different time points. Error bars are s.d., n=3.

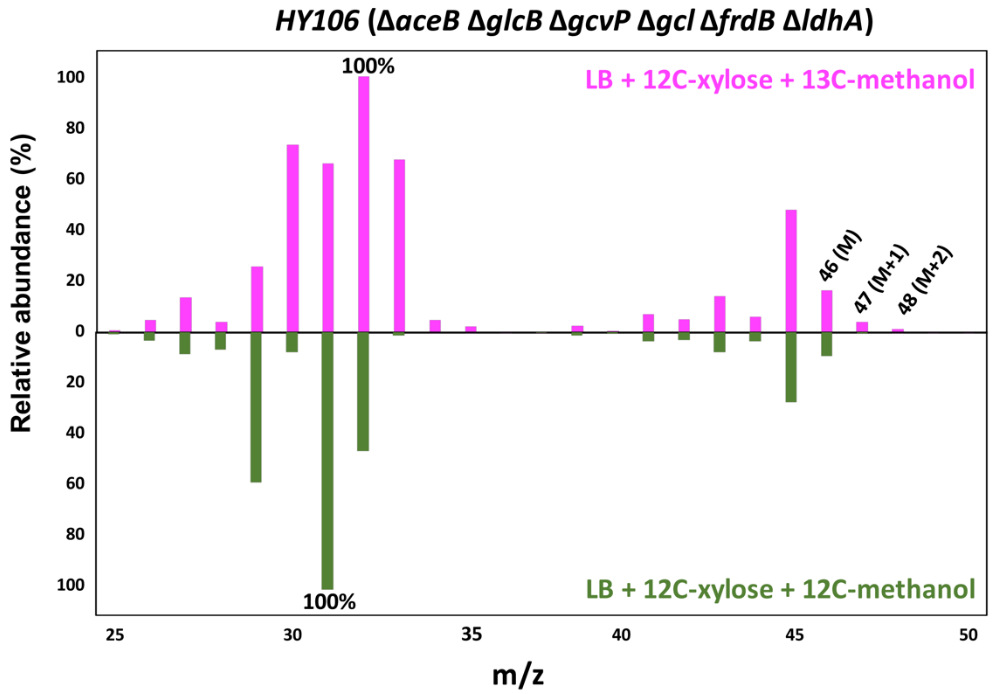

Supplementary Figure 10. The ethanol fractional pattern determined by gas chromatography-mass spectrometry (GC-MS). In the up part of the Figure (LB+12C-Xylose+13C-methanol), the fractional peaks of 34, 35, 47 and 48 (m/z) were detected, but were not appeared in the bottom part (LB+12C-Xylose+12C-methanol). A shifting in ethanol fractional peaks was observed in the engineered strain with expressing the complete cycle genes when incubation in 13C-methanol LB medium compared to the same engineered strain but supplied with unlabeled methanol. This result could be explained by 13C-methnaol incorporation into ethanol that caused the shifting in fractional peaks.

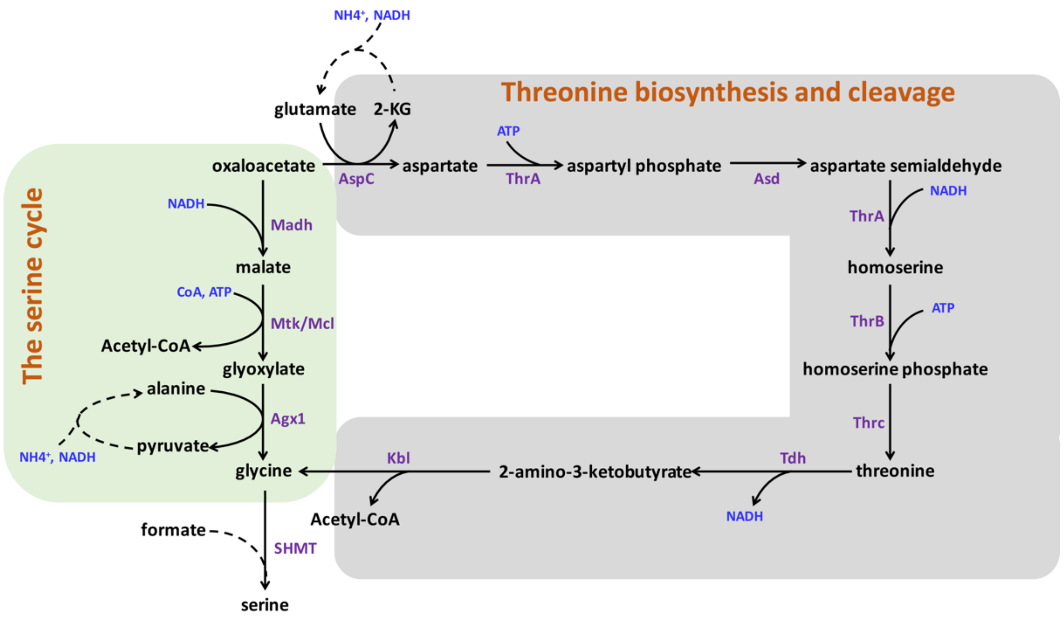

OAA to glycine and Ac-CoA	NADH	ATP	Enzymatic Steps
The serine cycle	2	1	4
The threonine route	2	2	8

Supplementary Figure 11. The threonine biosynthesis and cleavage route can convert oxaloacetate to glycine and acetyl-CoA with the expense of 2 ATP and 2 NADH.

Compared to Mtk/Mcl/Agx1 used in the modified serine cycle, the threonine biosynthesis and cleavage route includes more enzymatic steps and consumes one more ATP to produce glycine from OAA.

AspC: aspartate aminotransferase. ThrA: fused aspartate kinase/homoserine dehydrogenase. ThrB: homoserine kinase. ThrC: threonine synthase. Asd: aspartate-semialdehyde dehydrogenase.

Supplementary Table 1. Comparison among the C1-carbon assimilation cycles.

Name	C1 carbon assimilated	Cycle Product	Enzymes of the pathway	ATP equiv. for each acetyl-CoA synthesis	Implement in *E. coli*
The modified serine cycle	1. HCO_3^-/Formic acid<br>2. HCO_3^-/Methanol	Acetyl-CoA(C2)	11 (for formate)<br>13 (for methanol)	3 ATP	**Yes**. This study
The natural serine cycle (from *M. extorguens*)	1. HCO_3^-/Formic acid<br>2. HCO_3^-/Methanol	Acetyl-CoA(C2)	12 (for formate)<br>17 (for methanol)	2 ATP	No. Smejkalová et al., 2010
The RuMP cycle	Methanol	DHAP (C3)	9	2 ATP<br>(Type I methanotroph)	**Yes**. Whitaker et al., 2017
The MCC	Methanol	Acetyl-CoA(C2)	10	0 ATP	No. Bogorad et al., 2014
The CBB cycle	CO_2	3-p-glycerate (C3)	13	8 ATP	**Yes**. Antonovsky et al., 2016
The 3HP bicycle	HCO_3^-	Pyruvate (C3)	15	7 ATP	No. Mattozzi et al., 2013
The HP/HB cycle	HCO_3^-	Acetyl-CoA(C2)	13	6 ATP	No. Könneke et al., 2014
The rTCA cycle	CO_2	Pyruvate (C3)	9	2 ATP	No. Tang et al., 2010
The Wood-Ljungdahl pathway	CO_2	Pyruvate (C3)	9	1 ATP	No. Ragsdale et al., 2008

The natural serine cycle is the only pathway that can simultaneously assimilate one reduced C1 compound (formic acid or methanol) and one CO_2 equivalent to produce an acetyl-CoA (the C2 building block).

DHAP: dihydroxyacetone phosphate. The 3HP bicycle: the 3-hydroxypropionate bicycle. The HP/HB cycle: the 3-hydroxypropionate/4-hydroxybutyrate cycle.

Supplementary Table 2. Thermodynamic calculation of the modified serine cycle.

#	The modified serine cycle	$\Delta_r G'm$ (KJ/mol)
1	CO_2 + Phosphoenolpyruvate + H_2O <=> Orthophosphate + Oxaloacetate	-36.7
2	NADH + Oxaloacetate <=> NAD^+ + (S)-Malate	-30.3
3	ATP + CoA + (S)-Malate <=> ADP + Orthophosphate + Malyl-CoA	-6.6
4	Malyl-CoA <=> Acetyl-CoA + Glyoxylate	-4.2
5	L-Alanine + Glyoxylate <=> Pyruvate + Glycine	-8.3
6	NAD^+ + Methanol <=> NADH + Formaldehyde	34.2
7	NAD^+ + Formaldehyde + H_2O <=> NADH + Formate	-43.0
8	NAD^+ + Formate <=> NADH + CO_2	-14.1
9	ATP + Formate + Tetrahydrofolate <=> ADP + Orthophosphate + 10-Formyltetrahydrofolate	-4.8
10	10-Formyltetrahydrofolate <=> 5,10-Methenyltetrahydrofolate + H_2O	5.0
11	NADPH + 5,10-Methenyltetrahydrofolate <=> $NADP^+$ + 5,10-Methylenetetrahydrofolate	-9.9
12	Glycine + 5,10-Methylenetetrahydrofolate + H_2O <=> L-Serine + Tetrahydrofolate	6.5
13	L-Serine <=> NH_3 + Pyruvate	-45.8
14	ATP + Pyruvate + H_2O <=> Orthophosphate + AMP + Phosphoenolpyruvate	-13.3
**Total**	**HCO_3^- + Formate + 3NADH + 3ATP +CoA → Acetyl-CoA +AMP + 2ADP +3NAD^+**	**-148.4**
	**HCO_3^- + Methanol + NADH + 3ATP +CoA → Acetyl-CoA +AMP + 2ADP +NAD^+**	**-157.2**
	**2Formic acid + CoA +3ATP +2NADH → Acetyl-CoA + 3ADP + 2NAD^+**	**-162.5**
	**2Methanol + CoA +3ATP +2NAD^+ → Acetyl-CoA + 3ADP + 2NADH**	**-180.1**

The reaction Gibbs energy was calculated through eQuilibrator software. ΔrG'm(KJ/mol) is the free Gibbs energy when the reactant concentration was set to be 1 mM under the condition of pH=7 and 0.1 M ionic strength.

Supplementary Table 3. The net reactions of the modified serine cycle.

#	C1 Substrate(s)	The net reaction of the modified serine cycle
1	HCO_3^- and Formic acid	HCO_3^- + Formic acid + CoA +3ATP +3NADH → Acetyl-CoA + 3ADP + 3NAD
2	HCO_3^- and Methanol	HCO_3^- + Methanol + CoA +3ATP +NADH → Acetyl-CoA + 3ADP + NAD
3	2 Formic acid	2Formic acid + CoA +3ATP +2NADH → Acetyl-CoA + 3ADP + 2NAD
4	2 Methanol	2Methanol + CoA +3ATP +2NAD → Acetyl-CoA + 3ADP + 2NADH
5	2 Methanol	2Methanol +3ATP → Ethanol + 3ADP
6	HCO_3^- and 2 Formic acid	HCO_3^- + 2Formic acid + 4NADH + 4ATP + ubiquinone → pyruvate + ubiquinol
7	HCO_3^- and 2 Methanol	HCO_3^- + 2Methanol + 4ATP + ubiquinone → pyruvate + ubiquinol
8	3 Formic acid	3Formic acid + 3NADH + 4ATP + ubiquinone → pyruvate + ubiquinol
9	3 Methanol	3Methanol + 4ATP + 2NAD + ubiquinone → pyruvate + 2NADH + ubiquinol

Supplementary Table 4. Plasmids and strains used in the study.

Plasmids	Description	Reference
pCT20_S1	*ColE1 ori; Amp*^{R}*; PLlacO::His-medh(CT4-1)*	Wu, 2016.
pHY24	*ColA ori; Km*^{R}*; LacI; PLlacO1::mtkB(M.c) mtkA(M.c) mcl(M.e)*	This study
pHY49	*ColA ori; Km*^{R}*; LacI; PLlacO1::mtkB(M.c) mtkA(M.c) mcl(M.e) PLlacO1::madh(E.c) PLlacO1::pyc(C.g)*	This study
pHY56	*P15A ori; Amp*^{R}*; PLlacO1::sgaA(R.s)*	This study
pHY62	*P15A ori; Amp*^{R}*; PLlacO1::AGX1(S.c)*	This study
pHY64	*P15A ori; Amp*^{R}*; PLlacO1::sdaA(C.n)*	This study
pHY68	*P15A ori; Amp*^{R}*; PLlacO1::AGX1(S.c) sdaA(C.n)*	This study
pHY78	*P15A ori; Amp*^{R}*; PLlacO1::AGX1(S.c) PLlacO1::fthfl(M.t) mthfs(M.t)*	This study
pHY79	*P15A ori; Amp*^{R}*; PLlacO1::fthfl(M.t) mthfs(M.t)*	This study
pHY80	*P15A ori; Amp*^{R}*; PLlacO1::AGX1(S.c) PLlacO1::fthfl(M.t)*	This study
pHY81	*P15A ori; Amp*^{R}*; PLlacO1::sdaA(C.n) PLlacO1::fthfl(M.t) mthfs(M.t)*	This study
pHY84	*P15A ori; Amp*^{R}*; PLlacO1:: sdaA(C.n) AGX1(S.c) PLlacO1::fthfl(M.t) mthfs(M.t)*	This study
pHY86	*SCDF ori; Spec*^{R}*; PLlacO1::medh(CT4-1) faldh(P.p)*	This study
pHY87	*SCDF ori; Spec*^{R}*; PLlacO1::glyA(E.c) PLlacO1::medh(CT4-1) faldh(P.p)*	This study
pHY88	*SCDF ori; Spec*^{R}*; PLlacO1::glyA(E.c)*	This study
pHY89	*ColE1 ori; Gent*^{R}*; PLlacO1::pdup(S.e) adhB(Z.m)*	This study
pHY95	*P15A ori; Amp*^{R}*; PLlacO1::AGX1(S.c) PLlacO1::fthfl(M.e) folD(E.c)*	This study
**Strains**	**Relevant Genotype**	**Reference**
BW25113	*rrnBT14 ΔlacZWJ16 hsdR514 ΔaraBADAH33 ΔrhaBADLD78*	
HY93	Δ*serA* in *BW25113*	This study
HY96	Δ*serA* Δ*aceA* in *BW25113*	This study
HY94	Δ*serA gcvp::cat* in *BW25113*	This study
HY99	Δ*aceA* Δ*gcvp* in *BW25113*	This study
HY106	Δ*aceB* Δ*glcB* Δ*gcl* Δ*gcvp* Δ*frdB* Δ*ldhA* in *BW25113*	This study

Km^R: Kanamycin resistance; Amp^R: Ampicillin resistance; Spec^R: Spectinomycin resistance; Gent^R: Gentamycin resistance.

Supplementary Table 5. The ethanol fractional pattern determined by GC-MS.

m/z	Relative abundance (%)	
	12C-xylose + 13C-methanol	12C-xylose + 12C-methanol
25	1.01 ± 0.06	0.62 ± 0.07
26	5.16 ± 0.36	3.23 ± 0.27
27	13.88 ± 1.25	8.39 ± 1
28	4.32 ± 0.35	6.62 ± 0.64
29	25.94 ± 0.17	58.34 ± 5.3
30	73.25 ± 5.2	7.66 ± 0.6
31	66.16 ± 3.57	100
32	100	46.09 ± 3.87
33	67.52 ± 4.33	1.07 ± 0.07
34	4.96 ± 0.43	0
35	2.58 ± 0.11	0
37	0.16 ± 0.01	0
38	0.35 ± 0.02	0.17 ± 0.02
39	2.68 ± 0.14	1.11 ± 0.08
40	0.7 ± 0.08	0.43 ± 0.03
41	7.42 ± 0.58	3.32 ± 0.33
42	5.23 ± 0.25	2.93 ± 0.23
43	14.22 ± 1.27	7.75 ± 0.85
44	6.41 ± 0.41	3.52 ± 0.32
45	48.03 ± 4.22	27.16 ± 1.87
46 (M)	16.6 ± 0.9	12.19 ± 1.13
47 (M+1)	4.19 ± 0.26	0.34 ± 0.03
48 (M+2)	1.59 ± 0.09	0

Supplementary Table 6. Primers used in this study.

Primer name	Sequence
mtkA(M.c) F	5'-GATTCTGAAGGAGATATACCATGAATATCCATGAGTACCA-3'
mtkA(M.c) R	5'-GCTCATGGTATATCTCCTTTATCCCTTGACGATGGCGA-3'
mtkB(M.c) F	5'-AGAGGAGATATACCATGAGCGTATTCGTTAACAAG-3'
mtkB(M.c) R	5'-ATATTCATGGTATATCTCCTTCAGAATCTGATTCCGTGTT-3'
mcl(M.e) F	5'-CAAGGGATAAAGGAGATATACCATGAGCTTCACCCTGATCCA-3'
mcl(M.e) R	5'-CAAGCTTCTCGAGTTACTTTCCGCCCATCGCGT-3'
pyc(C.g) F	5'-GAGGAGATATACCATGTCGACTCACACATCTTCAA-3'
pyc(C.g) R	5'-CAAGCTTCTCGAGTTAGGAAACGACGACGATCAA-3'
madh(E.c) F	5'-GAGGAGATATACCATGAAAGTCGCAGTCCTCGG-3'
madh(E.c) R	5'-GGCCTCGTGATACGCCTTACTTATTAACGAACTCTT-3'
sdaA(C.n) F	5'-GAGGAGATATACCATGGCAGTCAGCGTCTTTGAT-3'
sdaA(C.n) R	5'-CGATACCGTCGACTTAGCATTCCACGATATTCA-3'
AGX1(S.c) F	5'-GAGGAGATATACCATGACTAAATCTGTAGATACG-3'
AGX1(S.c) R	5'-CTTATCGATACCGTCGACTCACTTTTTCCTCTGAAGAG-3'
fthfl(M.t) F	5'-GAGGAGATATACCATGTCCAAGGTACCCAGTGATA-3'
fthfl(M.t) R	5'-CTGGCATGGTATATCTCCTCTAGAAAAGACCGGTAATGA-3'
mthfs(M.t) F	5'-CTAGAGGAGATATACCATGCCAGCCCAGATCCTCGAC-3'
mthfs(M.t) R	5'-CGATACCGTCGACCTAGCGCCGGGCCGCCTCGACT-3'
glyA(E.c) F	5'-AAGAGGAGATATACCATGTTAAAGCGTGAAATGAA-3'
glyA(E.c) R	5'-GATACCGTCGACTTATGCGTAAACCGGGTAAC-3'

medh F	5’-GAGGAGATATACCATGACCCACCTGAACATCGCTA-3’
medh R	5’-CATGGTATATCTCCTTTACATCGCCGCAGCGAAGAT-3’
faldh(P.p) F	5’-GTAAAGGAGATATACCATGTCTGGCAATCGTGGAGTG-3’
faldh(P.p) R	5’-CGATACCGTCGACTTACGCCGCACCCCACATTTT-3’
Pdup(S.e) F	5’-GAGGAGATATACCATGAATACTTCTGAACTCGA-3’
Pdup(S.e) R	5’-GTATATCTCCTGCATGCTTAGCGAATAGAAAAGCCGTT-3’
adhB(Z.m) F	5’-CATGCAGGAGATATACCATGGCTTCTTCAACTTTTTAT-3’
adhB(Z.m) R	5’-CGATACCGTCGACTTAGAAAGCGCTCAGGAAGAGT-3’
sgaA(R.s) F	5’-GAGGAGATATACCATGTCGCTTGCGCACGGCCGT-3’
sgaA(R.s) R	5’-CTTATCGATACCGTCGACTCAGGCTGCCGCGCCGAGGCT-3’

The RBS sequence for expressing gene was 5’-AGGAGATATACC-3’.

In Supplementary Figure 2D, to regulate the expression levels of *ghrA*(*E.c*), three different RBS sequences were used as following:

RBS(H): 5’-AGGAGATATACC-3’,

RBS(M): 5’- CATCCGTTAATTACACACCCAGCAAACTACCTA-3’,

RBS(L): 5’- CAAGCCTACACAAATAACGACTTACCAACCT-3’.

Supplementary Table 7. Summary of enzymes used in the modified serine cycle.

Enzymes	EC #	Gene	Accession ID	Associated database	Origin
Phosphoenolpyruvate synthetase	2.7.9.2	*pps*	P23538	Biocyc	*E. coli*
Phosphoenolpyruvate carboxylase	4.1.1.31	*ppc*	P00864	Biocyc	*E. coli*
Pyruvate carboxylase	6.4.1.1	*pyc*	H7C7K2	Biocyc	*C. glutamicum*
Malate dehydrogenase	1.1.1.37	*mdh*	P61889	Biocyc	*E. coli*
Malate thiokinase	6.2.1.9	*sucCD2*	MCA1740<br>MCA1741	Biocyc	*M. capsulatus*
Malyl-CoA lyase	4.1.3.24	*mcl*	MexAM1_META1p1733	Biocyc	*M. extorquens*
Alanine-glyoxylate transaminase	2.6.1.44	*AGX1*	P43567	Biocyc	*S. cerevisiae*
Serine hydroxymethyltransferase	2.1.2.1	*glyA*	P0A825	Biocyc	*E. coli*
Serine dehydratase	4.3.1.17	*sdaA*	H16_A3622	Biocyc	*C. necator*
Formate tetrahydrofolate ligase	6.3.4.3	*moth_0109*	GH0A-119	Biocyc	*M. thermoacetica*
Methylene-tetrahydrofolate synthase	3.5.4.9	*moth_1516*	GH0A-1544	Biocyc	*M. thermoacetica*
Formaldehyde dehydrogenase	1.2.1.46	*fdhA*	PP_0328	Biocyc	*P. putida kt2440*
Methanol dehydrogenase	1.1.1.244	*medh*(*CT4-1*)	Wu *et al*., 2016		*C. necator*

Supplementary References

1. Förster, A.H., Gescher, J. Metabolic Engineering of *Escherichia coli* for Production of Mixed-Acid Fermentation End Products. Front. *Bioeng. Biotechnol.* **2**, 16 (2014).

2. Molina, I., Pellicer, M.T., Badia, J., Aguilar, J., Baldoma, L. Molecular characterization of *Escherichia coli* malate synthase G. Differentiation with the malate synthase A isoenzyme. *Eur. J. Biochem.* **224**, 541-548 (1994).

3. Chang, Y.Y., Wang, A.Y., Cronan JE Jr. Molecular cloning, DNA sequencing, and biochemical analyses of *Escherichia coli* glyoxylate carboligase. An enzyme of the acetohydroxy acid synthase-pyruvate oxidase family. *J. Biol. Chem.* **268**, 3911-3919 (1993).

4. Wu, T.Y., Chen, C.T., Liu, J.T., Bogorad, I.W., Damoiseaux, R., Liao, J.C. Characterization and evolution of an activator-independent methanol dehydrogenase from *Cupriavidus necator N-1. Appl. Microbiol. Biotechnol.* **100**, 4969–4983 (2016).

5. Smejkalová, H., Erb, T.J., Fuchs, G. Methanol assimilation in *Methylobacterium extorquens* AM1: demonstration of all enzymes and their regulation. *PLoS. One.* **5**, pii: e13001 (2010).

6. Whitaker, W.B., Jones, J.A., Bennett, R.K., Gonzalez, J.E., Vernacchio, V.R., Collins, S.M., Palmer, M.A., Schmidt, S., Antoniewicz, M.R., Koffas, M.A., Papoutsakis, E.T. Engineering the biological conversion of methanol to specialty chemicals in *Escherichia coli*. *Metab. Eng.* **39**, 49-59 (2017).

7. Bogorad, I.W., Chen, C.T., Theisen, M.K., Wu, T.Y., Schlenz, A.R., Lam, A.T., Liao, J.C. Building carbon-carbon bonds using a biocatalytic methanol condensation cycle. *Proc. Natl. Acad. Sci. U S A.* **111**, 15928-15933 (2014).

8. Antonovsky, N., Gleizer, S., Noor, E., Zohar, Y., Herz, E., Barenholz, U., Zelcbuch, L., Amram, S., Wides, A., Tepper, N., Davidi, D., Bar-On, Y., Bareia, T., Wernick, D.G., Shani, I., Malitsky, S., Jona, G., Bar-Even, A., Milo, R. Sugar Synthesis from CO_2 in *Escherichia coli*. *Cell.* **166**, 115-125 (2016).

9. Mattozzi, M.D., Ziesack, M., Voges, M.J., Silver, P.A., Way, J.C. Expression of the sub-pathways of the Chloroflexus aurantiacus 3-hydroxypropionate carbon fixation bicycle in *E. coli*: Toward horizontal transfer of autotrophic growth. *Metab. Eng.* **16**, 130-139 (2013).

10. Könneke, M., Schubert, D.M., Brown, P.C., Hügler, M., Standfest, S., Schwander, T., Schada von Borzyskowski, L., Erb, T.J., Stahl, D.A., & Berg, I.A. Ammonia-oxidizing archaea use the most energy-efficient aerobic pathway for CO_2 fixation. *Proc. Natl. Acad. Sci. U S A*. **111**, 8239-8244 (2014).

11. Tang, K.H., Blankenship, R.E. Both forward and reverse TCA cycles operate in green sulfur bacteria. *J. Biol. Chem*. **285**, 35848-54 (2010).

12. Ragsdale, S.W., Pierce, E. Acetogenesis and the Wood-Ljungdahl pathway of CO(2) fixation. *Biochim. Biophys. Acta*. **1784**, 1873-1898 (2008).
